# Exosome-mediated metabolic-immune regulatory axis: mechanisms of gastric cancer progression and resistance and targeting strategies

**DOI:** 10.3389/fimmu.2026.1915650

**Published:** 2026-09-07

**Authors:** Shun Yao, Hongyu Chai, Lulu Tang, Xin Li, Feifei Liu, Hai Jin, Biguang Tuo

**Affiliations:** 1 Department of Gastroenterology, Affiliated Hospital of Zunyi Medical University, Zunyi, Guizhou, China; 2 Digestive Disease Hospital, Affiliated Hospital of Zunyi Medical University, Zunyi, Guizhou, China; 3 Guizhou Clinical Research Center for Digestive Diseases, Zunyi, Guizhou, China; 4 Department of Gastroenterology, People’s Hospital of Dejiang County, Tongren, Guizhou, China; 5 Key Laboratory for Cancer Prevention and treatment of Guizhou Province, Zunyi, Guizhou, China

**Keywords:** chemoresistance, exosomes, gastric cancer, immune microenvironment, liquid biopsy, metabolic reprogramming, targeted therapy

## Abstract

Gastric cancer (GC) is characterized by marked heterogeneity and frequent resistance to chemotherapy and immunotherapy. Exosomes mediate intercellular communication between tumor, stromal, and immune cells and may couple metabolic adaptation to immune suppression. This review synthesizes evidence for a bidirectional exosome-mediated metabolic–immune axis in GC. Tumor-derived exosomes enhance glycolytic and other metabolic programs, remodel macrophage and lymphocyte function, and promote immune escape, metastasis, and treatment resistance. Conversely, exosomes released by cancer-associated fibroblasts, tumor-associated macrophages, and mesenchymal stromal cells reinforce tumor-cell metabolism, stemness, survival, and chemoresistance. Together, these reciprocal interactions form a self-reinforcing circuit that links metabolic plasticity with an immunosuppressive microenvironment. We further evaluate circulating exosomal cargoes as candidate biomarkers for liquid biopsy and discuss therapeutic approaches involving inhibition of exosome biogenesis or uptake, combined metabolic and immune targeting, and engineered exosome delivery. Finally, we emphasize the limitations imposed by model systems, extracellular-vesicle heterogeneity, incomplete cell-type specificity, and inconsistent isolation and characterization methods. Defining reproducible, subtype-specific exosome-mediated interactions will be essential for translating this framework into clinically useful biomarkers and therapeutic strategies.

## 1 Introduction

Gastric cancer (GC) remains a major global health burden, ranking fifth worldwide for both cancer incidence and cancer-related mortality, with approximately 980, 000 new cases and 642, 000 deaths estimated in 2024 (1). This high fatality rate is closely associated with factors such as low early diagnosis rates, a high propensity for metastasis, and potent therapeutic resistance (2, 3). GC is characterized by significant heterogeneity, with extensive variations across pathological types, molecular subtypes, and clinical phenotypes (4). Such profound heterogeneity leads to substantial disparities in treatment response among patients; consequently, the five-year survival rate for advanced GC remains dismal, particularly for those presenting with distant metastases, such as peritoneal or hepatic involvement, where the prognosis is even more severe (5–7). Furthermore, GC heterogeneity is reflected in the complexity of the tumor microenvironment (TME), which comprises malignant epithelial cells, non-hematopoietic stromal components such as cancer-associated fibroblasts (CAFs) and endothelial cells, and diverse hematopoietic immune populations, including tumor-associated macrophages (TAMs) and lymphocytes. Dynamic crosstalk among malignant cells, stromal components, and immune-cell populations collectively shapes GC progression and therapeutic resistance (8, 9).

Beyond rapid malignant progression, drug resistance represents another critical factor contributing to the poor prognosis of GC. While chemotherapy remains the cornerstone of systemic treatment for advanced GC, the efficacy of standard agents—represented by cisplatin and 5-fluorouracil (5-FU)—is often severely limited by the emergence of resistance (10–13). The development of such resistance involves diverse mechanisms, including increased drug efflux, enhanced DNA repair, evasion of apoptosis, and metabolic reprogramming (14). Concurrently, immune checkpoint inhibitors (ICIs) have offered new hope for GC therapy; however, clinical response rates remain modest, and immunotherapy resistance has emerged as a significant new challenge. Resistance to immune-checkpoint blockade arises through multiple, partly overlapping mechanisms. These include insufficient tumor-antigen presentation, exclusion or dysfunction of cytotoxic T cells, expansion of immunosuppressive myeloid and regulatory T-cell populations, and adaptive upregulation of inhibitory checkpoint pathways. These processes can also be reinforced by metabolic constraints within the TME, including hypoxia, glucose depletion, and accumulation of immunosuppressive metabolites (15, 16). Furthermore, chemoresistance and immunotherapy tolerance are frequently interconnected, culminating in multidrug resistance that further exacerbates the difficulty of clinical management.

The dynamic alterations within the TME serve as a critical hub for orchestrating GC progression and therapeutic resistance. These changes include regional hypoxia, nutrient depletion, extracellular acidification, matrix remodeling, and shifts in the abundance and functional states of stromal and immune cells. Rapid proliferation of tumor cells leads to localized hypoxia and nutrient deprivation, which in turn drives endogenous metabolic reprogramming—such as the Warburg effect and enhanced glutaminolysis—to provide the essential energy and substrates for proliferation and metastasis (5, 17–19). Simultaneously, this metabolic reprogramming modifies the composition of metabolites in the microenvironment, characterized by factors such as lactate accumulation and tryptophan depletion. These changes further “educate” or recruit immune cells, culminating in the formation of an immunosuppressive microenvironment (20, 21). During the development of this metabolically constrained and immunosuppressive microenvironment, extracellular vesicles (EVs)provide one route of intercellular communication by transferring nucleic acids, proteins, lipids, and metabolites among tumor, stromal, and immune cells (8, 9, 22). Such transfer can alter signaling and metabolic states in recipient cells, although its efficiency and cell-type specificity vary among experimental systems. Existing research has confirmed that exosomes not only transmit pro-progression signals—inducing epithelial-mesenchymal transition (EMT) and enhancing cancer stem cell (CSC) properties—but also mediate the horizontal transfer of drug-resistant phenotypes while remodeling the immune microenvironment to sustain both progression and resistance (23, 24). Metabolic reprogramming and immune dysfunction are therefore not independent features of GC. A major unresolved question is how EV-mediated communication couples tumor metabolism to immune remodeling and thereby contributes to progression and treatment resistance.

Although these processes have often been studied separately, their reciprocal interactions remain insufficiently synthesized. Therefore, this article will systematically analyze the core mechanisms of the exosome-mediated metabolic-immune axis in GC progression and chemoresistance. Furthermore, we will summarize relevant targeting strategies to provide a novel theoretical basis and therapeutic insights for overcoming the bottlenecks of chemoresistance in GC.

## 2 Biological characteristics of exosomes

### 2.1 Biogenesis and cargo loading

**Figure 1:**
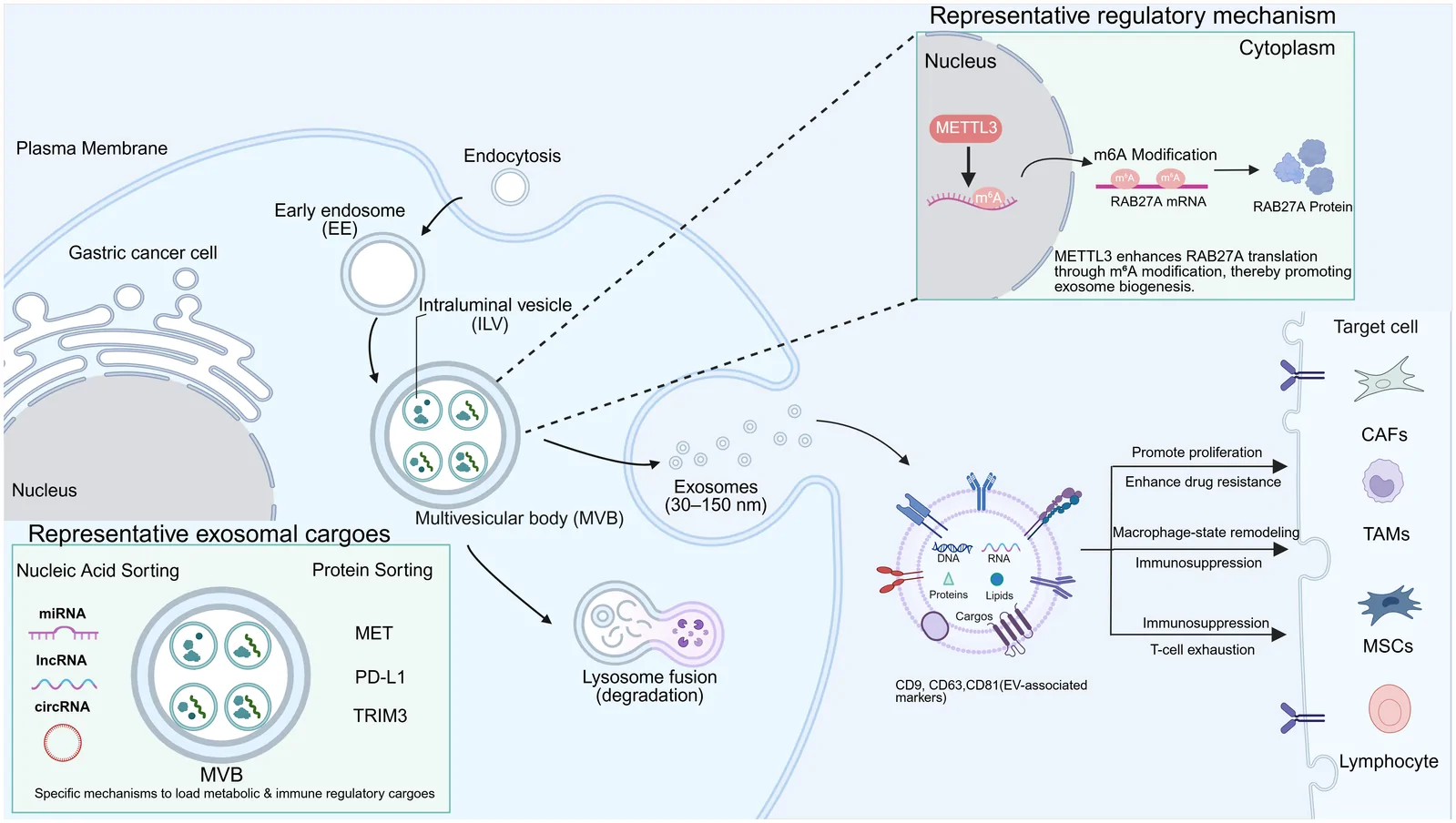
Exosome biogenesis, representative cargoes, and recipient-cell effects in the gastric cancer microenvironment. Exosomes originate as intraluminal vesicles within multivesicular bodies and are released following fusion of multivesicular bodies with the plasma membrane, whereas vesicles directed to lysosomes undergo degradation. In GC cells, METTL3-mediated m6A modification of RAB27A mRNA, together with YTHDF1-dependent recognition, enhances RAB27A translation and increases exosome production and release. Representative exosomal cargoes include miRNAs, lncRNAs, circRNAs, and proteins such as MET, PD-L1, and TRIM3. Following release, these vesicles can be taken up by GC cells and stromal or immune-cell populations, including CAFs, TAMs, MSCs, and lymphocytes, thereby modulating tumor-cell proliferation, treatment resistance, macrophage states, and antitumor immune responses. CD9, CD63, and CD81 are commonly assessed EV-associated proteins but are not individually specific for exosomes. CAF, cancer-associated fibroblast; circRNA, circular RNA; EV, extracellular vesicle; GC, gastric cancer; lncRNA, long non-coding RNA; miRNA, microRNA; MSC, mesenchymal stromal cell; MVB, multivesicular body; TAM, tumor-associated macrophage.

Exosomes are small EVs generated as intraluminal vesicles within multivesicular bodies (MVBs) and released into the extracellular space when MVBs fuse with the plasma membrane. They are commonly reported within an approximate size range of 30–150 nm and may contain proteins, lipids, metabolites, and several RNA species (25, 26). Exosome biogenesis begins with endosomal membrane invagination and intraluminal-vesicle formation, followed by MVB trafficking and fusion with the plasma membrane (25, 27). This process is regulated by various molecules. For instance, methyltransferase-like 3 (METTL3) can enhance the translation of the Ras-related protein Rab-27A (RAB27A) through m6A modification to promote exosome generation, whereas RAB27A point mutations significantly inhibit exosome secretion (28, 29). Exosomes possess a selective loading capacity for functional cargoes. Proteins can be specifically sorted into exosomes via post-translational modifications such as ubiquitination, phosphorylation, and glycosylation (30). The loading of nucleic acids (e.g., miRNAs, lncRNAs, and circRNAs) is closely associated with RNA-binding proteins, such as MBNL1 and hnRNPA1 (31, 32). Exosome delivery and uptake can occur through multiple mechanisms, including direct fusion with the recipient-cell membrane, receptor–ligand interactions at the cell surface, and internalization through endocytic or phagocytic pathways followed by intracellular cargo release (**Figure 1**) (26, 33, 34). These mechanisms support intercellular communication but do not, by themselves, demonstrate selective targeting of particular recipient-cell populations *in vivo*.

### 2.2 Cargo composition and exosome-mediated communication in the GC microenvironment

Among the nucleic acid cargoes investigated in GC-derived exosomes, miRNAs have been the most extensively studied. For instance, GC cell-derived exosomal miR-301a-3p, miR-21-5p, and miR-675-3p regulate metabolic adaptation, peritoneal dissemination, and immune escape through distinct recipient-cell and molecular pathways (**Table 1**) (35–37). LncRNAs, such as LINC00355, TTN-AS1, and SND1-IT1, participate in metabolic reprogramming and immune regulation through mechanisms such as competitive endogenous RNA (ceRNA) networks or by modulating gene transcription (38–40). Circular RNAs (circRNAs), including circPTBP3, circATP8A1, and circSHKBP1, leverage their high stability to form regulatory networks involved in metastasis, immune remodeling, and resistance (8, 41, 42). Protein cargoes consist of membrane markers, functional enzymes, and immunomodulatory proteins. Notable examples include PD-L1, which inhibits T-cell function; MET, which activates oncogenic signaling in recipient GC cells; and TRIM3, which exerts tumor-suppressive effects (43–45). In a non-GC experimental model, CAF-derived extracellular vesicles were shown to contain amino acids, lipids, and tricarboxylic acid-cycle intermediates and to support tumor-cell metabolism under nutrient-deprived conditions (46). Whether comparable metabolite transfer occurs through CAF-derived EVs in GC remains to be established.

**Table 1:** Exosomal cargo molecules involved in GC progression and resistance.

Molecular type	Cargo	Source cell/condition	Target cell	Downstream pathway	Biological effect	Clinical relevance	Refs
Protein	MET	Malignant ascites of advanced GC	GC cells	Oncogenic MET signaling	Transfers functional MET signaling and identifies MET-dependent therapeutic vulnerability	Patient-derived EVs may support selection and monitoring of MET-targeted therapy; prospective validation required	(43)
Protein	EMMPRIN	Peritoneal mesothelial cells	Diffuse-type GC cells	Receptor binding and MMP activation	Promotes extracellular-matrix degradation and invasion	Potential mediator of peritoneal invasion; mechanistic evidence	(52)
Protein	PD-L1	H. pylori CagA-expressing GC cells	CD8+ T cells	PD-1/PD-L1 inhibitory signaling	Suppresses T-cell proliferation, cytokine release, and cytotoxicity; promotes immune escape	Candidate immune-monitoring biomarker and therapeutic target	(53, 54)
Protein	TRIM3	GC patient serum	GC cells	SOX2 and EMT-related protein downregulation	Suppresses stemness and metastatic progression	Candidate circulating tumor-suppressive biomarker; validation required	(44)
Protein	YB-1	GC cells	Endothelial cells	Upregulation of VEGF and angiopoietin-1	Promotes endothelial activation and angiogenesis	Candidate anti-angiogenic target; preclinical evidence	(55)
Protein	PKM2	GC cells	Macrophages and MSCs	SCAP/SREBP1 lipid synthesis; NF-κB p65 acetylation	Promotes M2-like macrophage polarization and MSC-to-CAF differentiation	Pharmacologically modulated by mJPYZ in preclinical studies	(56–58)
Protein	CCT6A	CAFs	GC cells	β-catenin/c-Myc; suppression of DDIT4 and TXNIP	Enhances glycolysis, stemness, and chemoresistance	Candidate stromal metabolic target	(59)
Protein	Menin	CAFs	GC cells	HSPA6/JNK/JunD	Induces EMT and promotes GC-cell migration	Candidate CAF–tumor communication target; mechanistic evidence	(60)
Protein	UBR2	p53-deficient MSCs	GC cells	Wnt/β-catenin; CD44 and Cyclin D1 upregulation	Promotes growth, metastasis, and stemness	Candidate target of MSC-mediated tumor support	(61)
Protein	Wnt5a	Lymph-node-metastasis-derived GC cells	Bone-marrow MSCs	Wnt5a/YAP	Reprograms MSCs into a metastasis-supporting phenotype	Candidate mediator of lymph-node metastatic niche formation	(62)
Protein	TGF-β1	GC cells	Naive CD4+ T cells	FOXP3 and CTLA-4 induction	Promotes Treg differentiation and immune suppression	Candidate immunosuppressive cargo and therapeutic target	(63)
Protein	L-PGDS	MSC-derived EVs	GC cells	Inhibition of STAT3 phosphorylation	Reduces stemness	Preclinical therapeutic cargo; efficacy and biodistribution require validation	(64)
miRNA	miR-301a-3p	Hypoxic GC cells	GC cells	PHD3/HIF-1α positive-feedback loop	Enhances glycolytic reprogramming, progression, and metastasis	Candidate hypoxia-associated metabolic target; clinical biomarker performance requires separate validation	(35)
miRNA	miR-362-5p	H. pylori-infected gastric epithelial cells	GC cells	PTEN/AKT	Promotes GC-cell proliferation	Candidate infection-associated oncogenic cargo	(65)
miRNA	miR-21-5p	GC cells	peritoneal mesothelial cells	SMAD7/TGF-β/SMAD	Induces MMT and peritoneal dissemination	Candidate biomarker/target for peritoneal metastasis	(36)
miRNA	miR-519a-3p	GC cells	Hepatic macrophages	DUSP2/MAPK/ERK	Induces pro-angiogenic M2-like polarization and a liver pre-metastatic niche	Candidate target for preventing liver metastasis	(51)
miRNA	miR-675-3p	GC cells	GC cells and T cells indirectly	CXXC4/MAPK and PD-L1 upregulation	Promotes tumor immune escape	Candidate immune-evasion target	(37)
miRNA	miR-223-3p	N2 neutrophils	GC cells	FOXO3 and PI3K/AKT	Promote cancer-stem-cell properties and self-renewal	Candidate neutrophil–tumor stemness axis	(66)
miRNA	miR-425-5p	N2 neutrophils	GC cells	PTEN and PI3K/AKT	Promote cancer-stem-cell properties and self-renewal	Candidate neutrophil–tumor stemness axis	(66)
miRNA	miR-502-5p	GC cells	Endothelial cells/GC cells	Wnt/β-catenin inhibition	Suppresses angiogenesis and GC progression	Candidate tumor-suppressive cargo and therapeutic molecule	(67)
miRNA	miR-769-5p	Cisplatin-resistant GC cells	Cisplatin-sensitive GC cells	CASP9/p53	Transfers cisplatin resistance and promotes progression	Serum resistance biomarker candidate	(68)
miRNA	miR-501-5p	Doxorubicin-resistant GC cells	GC cells	BLID/caspase-9/-3 and AKT phosphorylation	Transfers anti-apoptotic and doxorubicin-resistant phenotypes	Candidate resistance biomarker/target	(69)
miRNA	miR-3681-3p	M2-polarized macrophages	GC cells	MLH1 suppression	Impairs mismatch repair and promotes cisplatin resistance	Candidate macrophage-derived resistance target	(70)
miRNA	miR-223	Macrophages	GC cells	Not established in the cited study	Transfers macrophage-mediated doxorubicin resistance to GC cells	Candidate target for macrophage-mediated chemoresistance	(71)
miRNA	miR-21 and miR-194	M2-polarized TAMs	GC cells	PTEN/PI3K/AKT	Inhibit cisplatin-induced apoptosis	Candidate TAM–tumor resistance targets	(72, 73)
miRNA	miR-522	CAFs	GC cells	ALOX15/lipid ROS/ferroptosis	Suppresses ferroptosis and promotes acquired chemoresistance	Candidate combination-therapy target	(32)
miRNA	miR-451	GC cells	Tumor-infiltrating T cells	AMPK suppression and mTOR activation	Promotes Th17-cell differentiation under glucose-deprived conditions	Candidate metabolic–immune feedback marker	(74)
miRNA	miR-27a	GC cells	Fibroblasts	CSRP2 suppression	Induces CAF conversion	Candidate tumor–CAF feedback target	(75)
miRNA	miR-1911-5p	TAMs	GC cells	MYB/AKR1B10; ferroptosis regulation	Inhibits ferroptosis and reinforces M2 polarization	Candidate positive-feedback resistance target	(76)
miRNA	let-7g-5p	SERPINE1-high GC cells	Macrophages	STAT3	Promotes M2-like polarization and a tumor-supportive microenvironment	Candidate stratified target in SERPINE1-high GC	(77)
miRNA	miR-92b-5p	GC cells	Macrophages	STAT3	Drives M2-like polarization and pro-invasive support	Candidate tumor–macrophage communication target	(78)
miRNA	miR-301b-3p	MSCs	GC cells	TXNIP suppression; P-gp/MRP induction	Promotes cisplatin/vincristine resistance and inhibits apoptosis	Candidate MSC-derived resistance target	(79)
miRNA	miR-221	GC tissue-derived MSCs	GC cells	Paracrine oncogenic signaling	Promotes proliferation and migration	Associated with lymph-node metastasis and TNM stage; biomarker potential	(80)
miRNA	miR-552-5p	Plasma EVs from patients with GC	NK cells	NKG2D and NKp30 downregulation	Suppresses perforin, granzyme, and IFN-γ production	Candidate circulating marker of NK-cell dysfunction	(81)
lncRNA	LINC00355	GC cells	GC cells	HDAC3/TP53INP1 transcriptional repression	Promotes EMT and metastasis	Candidate prognostic/therapeutic target	(38)
lncRNA	MALAT1	M2 TAMs	GC cells	β-catenin stabilization and HIF-1α upregulation	Enhances aerobic glycolysis, proliferation, metastasis, and resistance	Candidate TAM-derived metabolic/resistance target	(82)
lncRNA	CRNDE	TAMs	GC cells	PTEN degradation	Promotes cisplatin resistance	Candidate target for TAM-mediated chemoresistance	(83)
circRNA	circPTBP3	GC cells	Peritoneal mesothelial cells	TFAP2B/SGK1 transcription	Induces MMT and peritoneal metastasis	Candidate marker/target for peritoneal dissemination	(41)
circRNA	circATP8A1	GC cells	Macrophages	miR-1-3p/STAT6	Induces M2-like polarization and GC progression	Candidate tumor–macrophage communication target	(8)
circRNA	circSHKBP1	GC cells	Endothelial and GC cells	miR-582-3p/HuR/VEGF; HSP90 stabilization	Promotes angiogenesis and malignant progression	Serum diagnostic/prognostic candidate;	(42)
circRNA	circ_0001947	GC cells	CD8+ T cells	miR-661/miR-671-5p/CD39	Drives T-cell exhaustion and anti-PD-1 resistance	Candidate immunotherapy-resistance biomarker and target	(45)
circRNA	circKIAA1797	GC-cell-derived EVs	GC cells	miR-4429/PBX3	Regulates proliferation and glycolysis	Serum biomarker and metabolic target candidate	(84)

“Target cell” refers to the recipient-cell population in which EV uptake and/or cargo-dependent functional effects were experimentally evaluated. It does not imply exclusive or selective targeting in vivo.

### 2.3 Release and uptake characteristics in GC

Various cells within the GC microenvironment, including GC cells, CAFs, TAMs, and peritoneal mesothelial cells, are capable of secreting exosomes, and their release is significantly augmented under conditions such as hypoxia or chemotherapeutic stimulation (32, 41). For instance, hypoxia promotes the release of miR-301a-3p-rich exosomes from GC cells, while cisplatin treatment induces the secretion of apoptotic exosomes (35, 47). GC-derived exosomes are detectable in biofluids such as blood, ascites, and saliva and exhibit tissue-specific enrichment; specifically, malignant ascites-derived exosomes are enriched with MET, and serum exosomal circSHKBP1 levels correlate with GC progression (42, 43). Regarding uptake, GC cells, macrophages, and mesothelial cells have been reported to internalize exosomes through mechanisms such as endocytosis and membrane fusion. Surface molecules may influence this process; for example, CD44-related interactions and RGD modification have been associated with enhanced uptake by GC cells in experimental models (48, 49). GC-derived exosomes can remodel organ-specific pre-metastatic environments. In a liver-metastasis model, exosomal EGFR was taken up by liver stromal cells and suppressed miR-26a/b, thereby increasing HGF signaling and facilitating liver colonization (50). Other studies have reported uptake by hepatic macrophages or peritoneal mesothelial cells (41, 51). These findings support biologically consequential uptake in defined recipient populations, but they do not establish exclusive cell-type targeting or exclude broader systemic distribution.

## 3 Exosomes drive GC progression

### 3.1 Promotion of tumor cell proliferation and stemness maintenance

Exosomes lay the foundation for GC progression by transmitting pro-proliferative signals and enhancing CSC properties (85, 86). For example, exosomes secreted by *Helicobacter pylori*-infected gastric epithelial cells carry miR-362-5p, which targets and inhibits PTEN to activate the AKT pathway, thereby significantly promoting GC cell proliferation (65). Exosomes derived from malignant ascites are enriched with MET protein, which, upon uptake by tumor cells, activates the PI3K/AKT and MAPK pathways while upregulating c-Myc expression to enhance proliferative activity and anti-apoptotic capacity (43). Regarding stemness maintenance, N2 neutrophil-derived exosomes transfer miR-223-3p and miR-425-5p, which directly suppress FOXO3 and PTEN, respectively. Their coordinated effects activate PI3K/AKT signaling, enhance GC-cell stemness and metastatic potential, and reduce sensitivity to oxaliplatin (66). Additionally, EVs derived from GC stem cells can transfer lncRNA H19 to non-stem cancer cells, thereby enhancing stemness-associated phenotypes, tumorigenicity, and metastatic potential through activation of the YAP/CDX2 signaling axis (85).

### 3.2 Induction of EMT and disruption of tissue barriers

Epithelial-mesenchymal transition (EMT) and peritoneal mesothelial-to-mesenchymal transition (MMT) are pivotal steps in GC invasion and metastasis, processes regulated by various exosomal cargoes. GC cell-derived exosomal miR-21-5p is internalized by peritoneal mesothelial cells, where it targets SMAD7 and activates TGF-β/SMAD signaling. This paracrine interaction induces MMT, compromises the mesothelial barrier, and facilitates peritoneal dissemination (36). Furthermore, exosomal lncRNA LINC00355 promotes GC cell migration and distant metastasis by binding to HDAC3 to inhibit the transcription of TP53INP1 (38). When exosomal circPTBP3 from GC cells is internalized by peritoneal mesothelial cells, it recruits TFAP2B to the SGK1 promoter site, activating SGK1 transcription to induce MMT and promote peritoneal metastasis (41). Additionally, peritoneal mesothelial cell-derived exosomes carry extracellular matrix metalloproteinase inducer (EMMPRIN), which binds to surface receptors on diffuse-type GC cells to activate matrix metalloproteinases (MMPs). This leads to the degradation of the extracellular matrix, further enhancing the invasive potential of the tumor cells (52).

### 3.3 Mediation of angiogenesis

**Figure 2:**
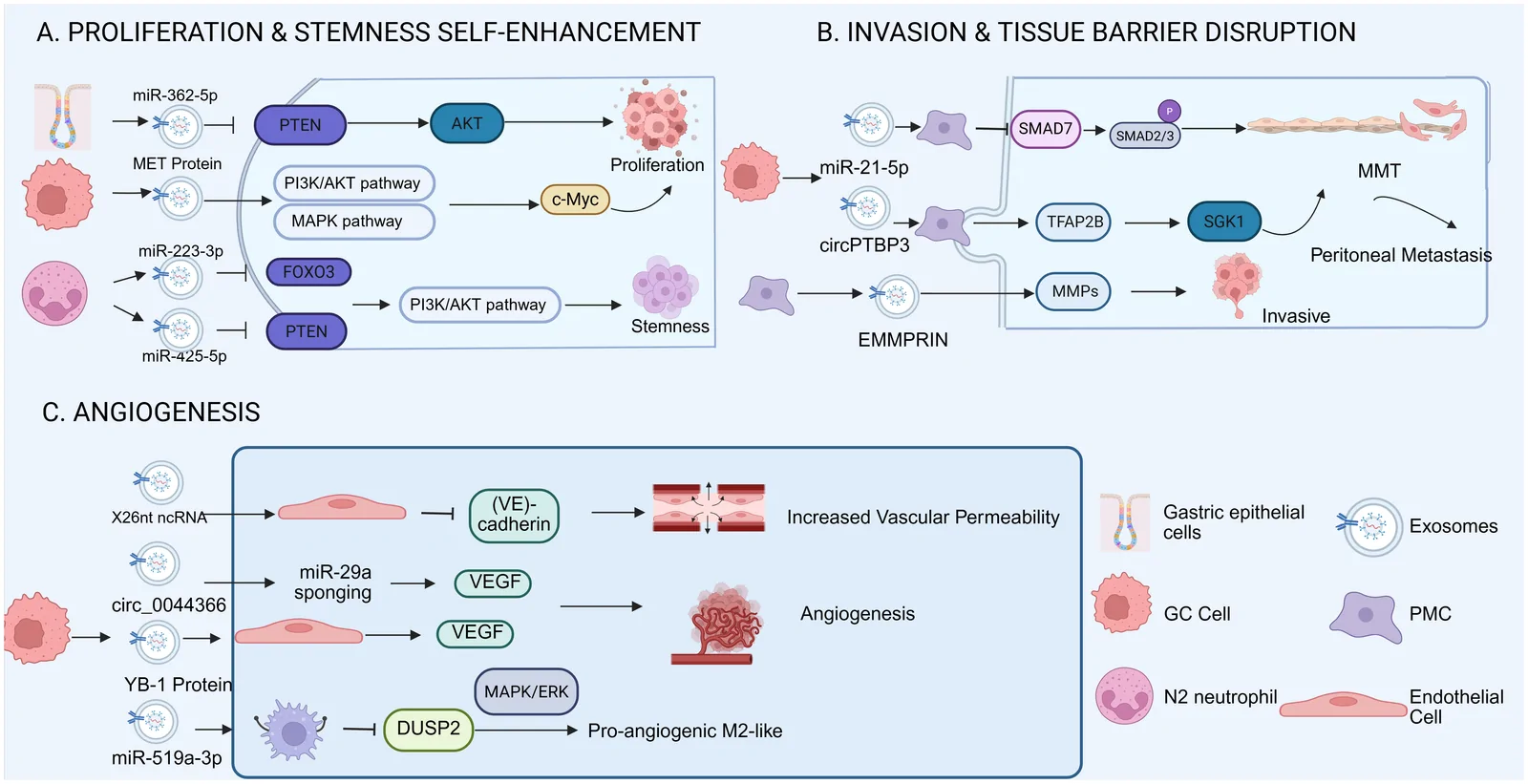
Exosome-mediated regulation of proliferation, stemness, invasion, tissue-barrier disruption, and angiogenesis in gastric cancer. **(A)** Exosomes released from H. *pylori*-infected gastric epithelial cells transfer miR-362-5p to GC cells, suppressing PTEN and activating AKT signaling. Malignant-ascites-derived exosomal MET activates PI3K/AKT and MAPK–c-Myc signaling in recipient GC cells. N2 neutrophil-derived exosomal miR-223-3p and miR-425-5p directly suppress FOXO3 and PTEN, respectively, resulting in coordinated activation of PI3K/AKT signaling and enhancement of GC-cell stemness. **(B)** GC-derived exosomal miR-21-5p is internalized by peritoneal mesothelial cells, where it suppresses SMAD7 and activates TGF-β/SMAD signaling to induce mesothelial-to-mesenchymal transition. Exosomal circPTBP3 activates the TFAP2B–SGK1 pathway in peritoneal mesothelial cells, whereas peritoneal-mesothelial-cell-derived EV-associated EMMPRIN activates MMPs in diffuse-type GC cells. These pathways compromise the peritoneal barrier and promote invasion and peritoneal dissemination. **(C)** GC-derived exosomal X26nt increases vascular permeability by reducing endothelial VE-cadherin. Exosomal circ_0044366 sponges miR-29a and increases VEGF expression, while exosomal YB-1 enhances the expression of VEGF and angiopoietin-1 in endothelial cells. In addition, exosomal miR-519a-3p suppresses DUSP2 in hepatic macrophages, activates MAPK/ERK signaling, and induces a proangiogenic M2-like macrophage state. EMT, epithelial–mesenchymal transition; GC, gastric cancer; MMP, matrix metalloproteinase; MMT, mesothelial-to-mesenchymal transition; PMC, peritoneal mesothelial cell; VE-cadherin, vascular endothelial cadherin; VEGF, vascular endothelial growth factor.

Exosome-driven angiogenesis is a critical link in GC progression. GC cells release exosomes to deliver functional molecules to endothelial cells and various stromal cells within the TME, directly or indirectly activating pro-angiogenic signals to support tumor growth and distant metastasis (87).Research indicates that a 26-nt-long ncRNA (X26nt), produced by cellular stress, is significantly elevated in GC and its exosomes. This molecule can be delivered via exosomes to human umbilical vein endothelial cells, where it directly binds to and downregulates the expression of vascular endothelial (VE)-cadherin. This process increases vascular permeability, thereby promoting angiogenesis and tumor growth (87). Additionally, circ_0044366 is highly expressed in GC-derived exosomes and influences the proliferation, migration, and tube formation of endothelial cells by acting as a sponge for miR-29a (88). Beyond direct effects on endothelial cells, GC exosomes indirectly promote angiogenesis by modulating the TME. For example, GC-derived exosomes can deliver miR-519a-3p to hepatic macrophages, targeting DUSP2 to activate the MAPK/ERK pathway. This induces the polarization of macrophages toward a pro-angiogenic M2-like phenotype, establishing a vascularized pre-metastatic niche in the liver (51). Furthermore, exosomal Y-box binding protein 1 (YB-1) has been identified as a key component of pro-angiogenic function, as it upregulates the expression of various pro-angiogenic factors, such as vascular endothelial growth factor (VEGF) and angiopoietin-1, in endothelial cells (55). Conversely, exosomal miR-502-5p has been found to inhibit GC angiogenesis by repressing the Wnt/β-catenin pathway, highlighting the complexity and potential tumor-suppressive roles of exosomal cargoes in vascular regulation (67). At the therapeutic level, engineered exosomes have demonstrated potential as delivery systems. For instance, exosomes loaded with HGF siRNA can be effectively delivered to tumor sites, significantly inhibiting both tumor and vascular growth (89). Collectively, these mechanisms constitute a complex exosome-mediated vascular regulatory network, providing novel targets for the treatment of GC (**Figure 2**).

## 4 Exosome-mediated chemoresistance in GC

### 4.1 Direct transmission of resistance signals

Exosomes can directly transmit resistance-associated molecules between cells, enabling sensitive cells to acquire a resistant phenotype. In the context of chemotherapy, exosomes secreted by cisplatin-resistant GC cells are enriched with miR-769-5p, which targets and inhibits CASP9 to block apoptotic pathways while promoting the degradation of p53, thereby allowing sensitive cells to tolerate cisplatin-induced damage (68).Exosomes from doxorubicin-resistant cells carry miR-501-5p, which downregulates BLID, leading to the inactivation of the caspase-9/-3 pathway and the activation of Akt phosphorylation, thus transmitting anti-apoptotic and resistant phenotypes (69). Additionally, M2-type TAM-derived exosomes containing circ_0008253 promote oxaliplatin efflux and reduce intracellular drug concentrations by upregulating ABCG2 expression (90). Conversely, exosomal delivery of miR-107 has been shown to significantly enhance the sensitivity of resistant GC cells to chemotherapeutic agents via the HMGA2/mTOR/P-gp axis (91).

Regarding immunotherapy resistance, GC cell-derived exosomes carry PD-L1, which binds to PD-1 on the surface of CD8^+^ T cells to directly inhibit their activity. This mechanism operates independently of the tumor cell’s own PD-L1 expression, establishing a “non-cell-autonomous” form of tolerance (54, 92).

### 4.2 Regulation of DNA repair and apoptotic pathways

M2-polarized macrophage-derived exosomal miR-3681-3p promotes cisplatin resistance by targeting MLH1 (70). Macrophage-derived exosomal miR-223 has also been reported to confer doxorubicin resistance (71).In terms of apoptotic regulation, exosomes derived from M2-polarized TAMs deliver miR-21 and miR-194 to downregulate PTEN expression and activate the PI3K/AKT pathway, which subsequently inhibits cisplatin-induced apoptosis (72, 73).

## 5 Exosome-mediated bidirectional metabolic-immune crosstalk

### 5.1 Tumor metabolites shape immunosuppression via exosomes

Following exosome-associated metabolic reprogramming, GC cells produce glucose metabolites, amino acid intermediates, and lipid molecules. These metabolites, together with exosomal nucleic acids and proteins, can influence innate and adaptive immune cells following exosome release and uptake. Such communication may inhibit anti-tumor immune functions and contribute to the establishment of an immunosuppressive microenvironment, thereby linking the metabolic phenotype of GC cells to immune dysfunction.

#### 5.1.1 Glucose competition and lactate accumulation

Enhanced aerobic glycolysis is a prominent metabolic feature of GC and contributes to the establishment of an immunosuppressive microenvironment through increased glucose consumption and lactate production. Exosomes can further reinforce glycolytic activity in recipient cells by transferring regulatory molecules. Under hypoxic conditions, GC cells release exosomes enriched in miR-301a-3p. Following uptake by neighboring GC cells, miR-301a-3p targets PHD3 and prevents HIF-1α degradation, thereby increasing the expression of glycolysis-related proteins, including GLUT1, HK2, and LDHA. This positive feedback loop enhances glucose uptake and lactate production and promotes tumor growth and metastasis (35).

The high glucose demand of GC cells reduces glucose availability to tumor-infiltrating lymphocytes, impairing the metabolic activity, proliferation, cytokine production, and effector functions of CD8^+^ T cells. CD155/TIGIT signaling further aggravates this metabolic competition, whereas glucose supplementation or TIGIT blockade partially restores CD8^+^ T-cell function (93). Lactate produced by highly glycolytic GC cells also contributes to immune suppression. Lactate activates GPR81 signaling in GC cells, induces CX3CL1 expression, and promotes regulatory T-cell (Treg) recruitment. In a humanized GC model, GPR81 deficiency reduced Treg infiltration and restored CD8^+^ T-cell activity, indicating that the lactate/GPR81/CX3CL1 axis contributes to immune tolerance in highly glycolytic GC (94).

Exosomal miR-451 provides a more direct link between glucose availability and immune-cell differentiation. Under glucose-deprived conditions, miR-451 is transferred from GC cells to tumor-infiltrating T cells via exosomes. In recipient T cells, miR-451 suppresses AMPK activity, enhances mTOR signaling, and promotes T helper 17 (Th17) cell differentiation. Elevated levels of circulating exosomal miR-451 in patients with GC have also been associated with increased Th17-cell frequencies and poor postoperative outcomes (74).

Collectively, exosomal signaling and soluble metabolites generated by glycolysis cooperate in reshaping the GC immune microenvironment. Exosomal miRNAs participate in the glycolytic adaptation of tumor cells and glucose-sensitive immune responses, whereas extracellular lactate promotes Treg recruitment and suppresses CD8^+^ T-cell function through GPR81-associated signaling. Nevertheless, there is currently no direct evidence that GC-derived exosomes transport lactate.

#### 5.1.2 Amino acid deprivation and accumulation of immunoregulatory metabolites

Amino acid metabolism is extensively reprogrammed in GC, supporting tumor growth, redox homeostasis, macromolecular biosynthesis, and immune regulation. GC cells commonly increase glutamine uptake to replenish tricarboxylic acid cycle intermediates and sustain nucleotide, protein, and lipid synthesis. The glutamine transporter ASCT2 and glutamine synthetase are expressed in GC cells, and inhibition of either protein suppresses tumor-cell growth, indicating that GC cells exhibit a degree of dependence on glutamine metabolism (95). Because activated lymphocytes also require glutamine to maintain their proliferation and effector functions, increased glutamine consumption by GC cells may intensify nutrient competition within the tumor microenvironment. However, direct evidence that glutamine consumption by GC cells causes immune-cell dysfunction remains limited.

Tryptophan catabolism provides a more clearly defined connection between amino acid metabolism and immune regulation in GC. IDO1 catalyzes the conversion of tryptophan into kynurenine, thereby reducing local tryptophan availability while generating immunoregulatory metabolites. In GC tissues, IDO expression is positively associated with Foxp3^+^ Treg infiltration and the abundance of tumor-infiltrating DC2 cells, but negatively associated with the intratumoral CD4^+^/CD8^+^ T-cell ratio. IDO expression, dendritic-cell subset distribution, and Foxp3^+^ Treg infiltration have also been associated with tumor stage and patient prognosis (96). Clinical metabolomic analyses have further revealed reduced circulating tryptophan levels in patients with GC, particularly in those with advanced disease, supporting the dysregulation of the tryptophan–kynurenine pathway during GC progression (97). Mechanistically, tryptophan depletion can restrict effector T-cell proliferation, whereas kynurenine activates aryl hydrocarbon receptor (AhR) signaling and promotes Treg differentiation. In a non-tumor-specific experimental system using human plasmacytoid dendritic cells, IDO inhibition reduced Treg generation, whereas the addition of exogenous kynurenine restored this effect (98). These findings provide a mechanistic basis for the regulation of dendritic cell–Treg interactions by the IDO–kynurenine pathway, although its direct relevance to GC requires further validation.

Exosomal signaling may represent another mechanism linking GC cells to amino acid-sensitive immune responses. Exosomes released by Epstein–Barr virus-associated GC cells suppress the maturation of monocyte-derived dendritic cells, as indicated by reduced CD86 expression (99). However, the specific exosomal cargo responsible for this effect was not identified, and the study did not demonstrate the exosomal delivery of IDO1, kynurenine, or other amino acid metabolites. Thus, dysregulated amino acid metabolism and exosome-mediated dendritic-cell suppression should currently be regarded as potentially interconnected processes for which a direct causal relationship has yet to be established.

#### 5.1.3 Lipid metabolism-mediated regulation of immune cell function

Lipid metabolic reprogramming is an important component of GC immune-microenvironment remodeling and influences the differentiation and function of multiple immune-cell populations. Macrophages exhibit considerable metabolic plasticity, and their functional states can change in response to alterations in lipid synthesis and utilization. GC-derived exosomal PKM2 provides direct evidence that tumor cells can reprogram macrophage lipid metabolism (56).

Following its uptake by macrophages, GC-derived exosomal PKM2 suppresses SCAP polyubiquitination and promotes the nuclear translocation of SREBP1. Activation of the SCAP/SREBP1 pathway increases the expression of lipogenic enzymes, including FASN, ACACA, and ACLY, and induces a tumor-promoting M2-like macrophage phenotype. These metabolically reprogrammed macrophages subsequently enhance the proliferation, migration, and invasion of GC cells. Plasma exosomal PKM2 levels are elevated in patients with GC and are associated with advanced disease and poor survival, suggesting its potential value as a biomarker (56). Nevertheless, its diagnostic and prognostic performance requires validation in independent, multicenter cohorts.

Abnormal lipid metabolism also affects immune-cell populations other than macrophages. In several tumor models, lipid accumulation impairs antigen processing and presentation by dendritic cells, whereas fatty acid synthesis and oxidation support the expansion and immunosuppressive activity of tumor-infiltrating Tregs (100–102). In addition, CD36-mediated fatty acid uptake increases lipid peroxidation and ferroptosis in tumor-infiltrating CD8^+^ T cells, thereby reducing cytotoxic cytokine production and antitumor activity (103). These mechanisms, however, have primarily been characterized in non-GC tumor models, and GC-specific evidence linking these immunometabolic alterations to defined exosomal cargo remains lacking. Current evidence for exosome-mediated lipid–immune regulation in GC is therefore largely confined to PKM2-driven lipogenesis and M2-like polarization in macrophages.

### 5.2 Immune microenvironment feedbacks to tumor metabolism via exosomes

**Figure 3:**
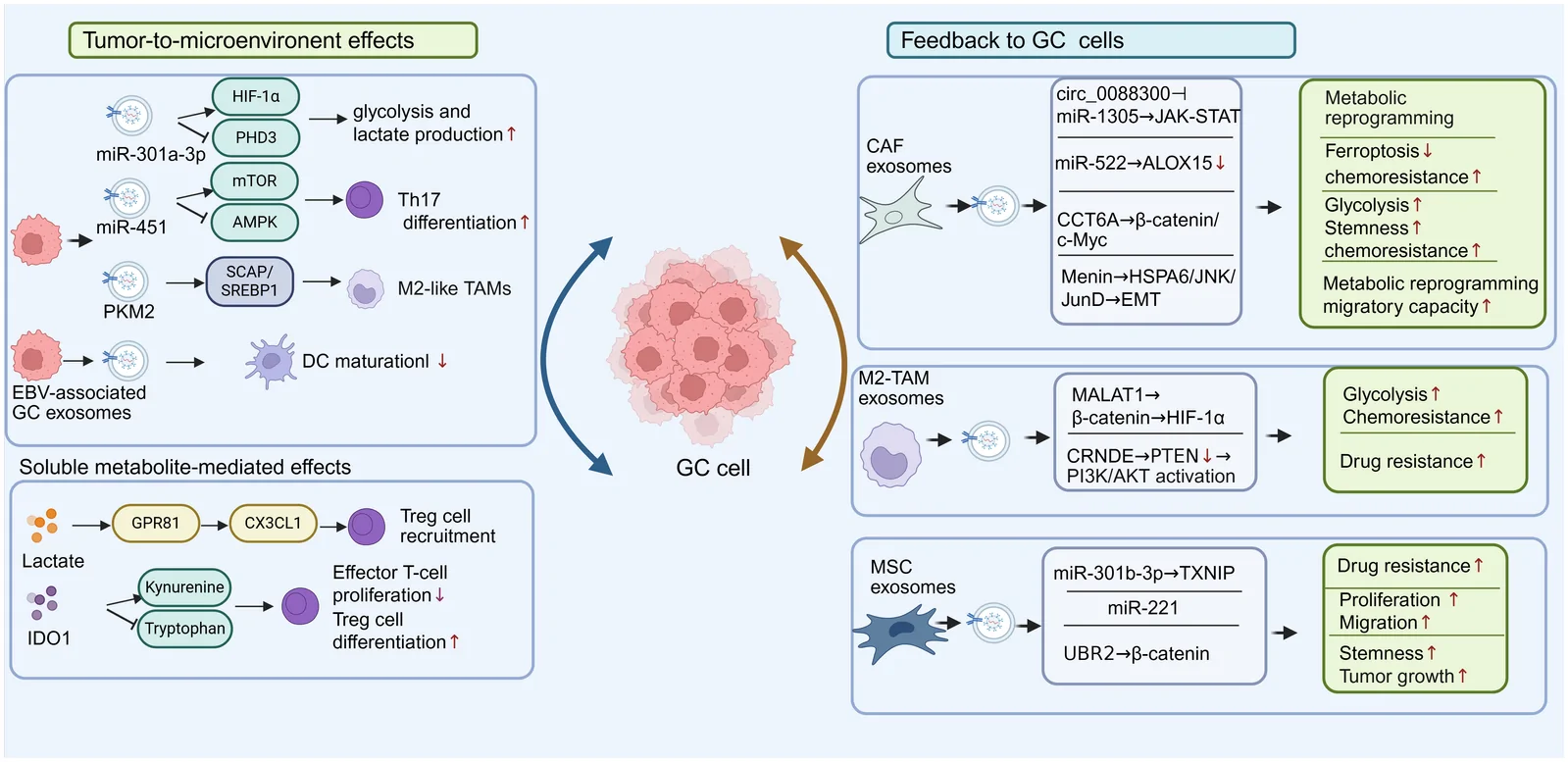
Bidirectional exosome-associated metabolic–immune crosstalk in gastric cancer. Tumor-to-microenvironment signaling is shown on the left. Hypoxic GC-derived exosomal miR-301a-3p suppresses PHD3 and stabilizes HIF-1α in recipient GC cells, thereby reinforcing glycolysis and lactate production. Exosomal miR-451 suppresses AMPK and activates mTOR signaling in tumor-infiltrating T cells, promoting Th17 differentiation under glucose-deprived conditions. GC-derived exosomal PKM2 activates the SCAP/SREBP1 lipogenic program in macrophages and promotes a tumor-supportive M2-like state, whereas EBV-associated GC exosomes inhibit dendritic-cell maturation through cargoes that remain incompletely defined. Soluble metabolic signals act in parallel with exosomal communication: lactate activates the GPR81–CX3CL1 pathway and recruits Tregs, whereas IDO1-mediated tryptophan catabolism reduces tryptophan availability and increases kynurenine-dependent immunoregulatory signaling. Microenvironment-to-tumor feedback is shown on the right. CAF-derived circ_0088300, miR-522, CCT6A, and menin regulate JAK/STAT signaling, ferroptosis, glycolysis, stemness, chemoresistance, and migration. M2-like-TAM-derived MALAT1 and CRNDE enhance glycolytic adaptation and drug resistance through β-catenin/HIF-1α and PTEN/PI3K/AKT signaling, respectively. MSC-derived exosomal cargoes further influence proliferation, stemness, drug resistance, and stromal remodeling. Together, these reciprocal pathways form an exosome-associated metabolic–immune feedback circuit in GC. CAF, cancer-associated fibroblast; DC, dendritic cell; EBV, Epstein–Barr virus; GC, gastric cancer; IDO1, indoleamine 2, 3-dioxygenase 1; MSC, mesenchymal stromal cell; TAM, tumor-associated macrophage; Treg, regulatory T cell.

Immune and stromal cells reprogrammed within the TME can, in turn, release exosomes that transmit metabolic regulatory signals to GC cells. This reciprocal communication reinforces tumor-cell metabolic adaptation, proliferation, metastasis, and treatment resistance, thereby establishing a bidirectional link between immune remodeling and tumor metabolism (**Figure 3**). Although extracellular vesicles may contain metabolites, direct delivery of metabolically active substrates by stromal- or immune-cell-derived vesicles has not been consistently demonstrated in GC and should therefore be interpreted cautiously.

#### 5.2.1 CAFs

As core interstitial cells of the TME, CAFs not only regulate tumor progression by remodeling the extracellular matrix and secreting inflammatory factors but also provide metabolic replenishment and phenotypic regulation for tumor cells through exosome-mediated intercellular communication. This supports tumor survival, proliferation, and drug resistance in nutrient-depleted microenvironments. This metabolic synergy is not merely simple material transport; rather, it involves the delivery of functional molecules via exosomes to achieve precise metabolic reprogramming of tumor cells while constructing a bidirectional regulatory loop.

CAF-derived exosomes can regulate tumor metabolic pathways by delivering functional nucleic acids. In lung adenocarcinoma, CAF-specific lncRNA LINC01614 is transported to tumor cells via exosomes, where it binds to ANXA2 and p65 to activate NF-κB signaling. This upregulates the expression of glutamine transporters SLC38A2 and SLC7A5, enhancing glutamine metabolism in tumor cells. Conversely, tumor-derived pro-inflammatory cytokines can upregulate LINC01614 expression in CAFs, forming a feed-forward loop (104). In GC, CAF exosomal circ_0088300, driven by KHDRBS3, is packaged and transported to GC cells. It acts as a sponge for miR-1305 to activate JAK/STAT signaling, accelerating metabolic reprogramming during proliferation and migration (105). Additionally, CAF-derived exosomal miR-522 targets ALOX15 to inhibit lipid-ROS accumulation and ferroptosis, thereby reducing sensitivity to chemotherapy-induced metabolic stress. This process depends on the USP7/hnRNPA1 axis to regulate the packaging and secretion of miR-522 (32). Furthermore, CAF exosomal lncRNA DACT3-AS1 mediates ferroptosis through the miR-181a-5p/SIRT1 axis to reverse oxaliplatin resistance, highlighting the dual role of exosomal nucleic acids in regulating both metabolism and drug resistance (106).

CAF-derived exosomes can directly provide metabolic substrates to tumor cells, thereby alleviating the pressure of nutrient deprivation. Exosomes secreted by patient-derived CAFs (CDEs) contain a complete range of metabolites, including amino acids, lipids, and tricarboxylic acid (TCA) cycle intermediates. Upon uptake by tumor cells, these cargoes directly participate in central carbon metabolism. ^13^C-labeled isotope tracing experiments have confirmed that CDEs can deliver amino acids to nutrient-deficient tumor cells while simultaneously inhibiting mitochondrial oxidative phosphorylation. This promotes glycolysis and glutamine-dependent reductive carboxylation, ultimately forming a metabolic phenotype adapted to the TME (46, 107).

Protein molecules within CAF exosomes also participate in the regulation of tumor metabolism. In GC, CAFs transmit CCT6A protein to tumor cells via exosomes; this protein interacts with β-catenin and induces its phosphorylation and nuclear translocation. Through c-Myc activation, this process inhibits the glycolytic inhibitors DDIT4 and TXNIP, thereby enhancing tumor cell stemness, chemoresistance, and glycolytic levels. Furthermore, the regulatory loop formed between miR-922 and c-Myc stabilizes CCT6A expression (59). Menin protein is also delivered to GC cells via CAF exosomes, activating the HSPA6/JNK/JunD pathway and inducing EMT, which synchronously regulates metabolic reprogramming and migratory capacity (60). Conversely, GC cell-derived exosomes can transfer PKM2 to mesenchymal stem cells (MSCs), inducing their differentiation into CAFs. During this differentiation, nuclear PKM2 associates with NF-κB p65 acetylation, promoting the transcription of inflammatory factors such as IL-6 and IL-8, which in turn feeds back to support tumor cell metabolism and proliferation (57).

Metabolic synergy between CAFs and tumor cells relies on bidirectional regulatory loops to maintain stability. For instance, GC cell-derived exosomal miR-27a induces the transformation of fibroblasts into CAFs. These transformed CAFs exert pleiotropic effects that enhance the malignant behavior and metabolic activity of GC cells, with CSRP2—a downstream target of miR-27a—showing significant downregulation that promotes GC cell proliferation and metabolic reprogramming (75). This bidirectional regulation ensures the continuous activation of the metabolic replenishment function of CAFs, making it a core component in the maintenance of tumor metabolic homeostasis.

#### 5.2.2 TAMs

TAMs account for 30%–50% of the immune cells within the GC TME, and their functional plasticity makes them a central hub of the immune-metabolic regulatory network (108). In advanced GC, TAMs predominantly exhibit an M2-like phenotype and deliver molecules such as nucleic acids and proteins to tumor cells via exosome-mediated intercellular communication. This regulates the metabolic phenotype of tumor cells while reinforcing their own pro-tumor characteristics through metabolic signal feedback, forming a “TAMs-exosome-tumor metabolism” synergistic network (108, 109).

At the nucleic acid level, M2-type TAM-derived exosomes are enriched with lncRNA MALAT1, which, upon delivery to GC cells, activates signaling pathways by stabilizing β-catenin and upregulating HIF-1α. This enhances aerobic glycolysis and promotes proliferation, metastasis, and chemoresistance (82). Additionally, lncRNA CRNDE promotes the degradation of PTEN to weaken its inhibition of metabolic pathways, thereby enhancing cisplatin resistance (83). Furthermore, miR-1911-5p targets the MYB/AKR1B10 axis to inhibit ferroptosis while feedback-promoting M2 polarization of macrophages, establishing a positive feedback loop (76).

At the protein level, GC cell-derived exosomes can deliver PKM2 to macrophages, inducing their polarization toward the M2 phenotype. These polarized TAMs then provide feedback to GC cells via exosomal or paracrine signals to support metabolic reprogramming (56). Exosomal PKM2 can enhance lipid synthesis in TAMs by stabilizing the SCAP protein, triggering SREBP1 nuclear translocation and the expression of fatty acid synthase, which effectively remodels their metabolic phenotype (56). Notably, modified Jianpi Yangzheng(mJPYZ) can reduce the delivery of exosomal PKM2, thereby inhibiting M2-type TAM differentiation and its pro-tumor metabolic effects (58).

This regulation is bidirectional, as tumor cells act upon TAMs via exosomes to form a regulatory loop. For instance, GC cells with high SERPINE1 expression secrete exosomes rich in let-7g-5p, which induces M2 polarization of macrophages through STAT3 signaling, forming a feed-forward amplification loop that optimizes the microenvironment to support tumor metabolism (77). Additionally, GC cells regulate the secretion of exosomal miR-92b-5p through the PLXNC1 signaling axis; once this miRNA enters macrophages, it activates STAT3 to drive M2 polarization and provide pro-invasive metabolic support (78).

#### 5.2.3 MSCs

As a critical stromal component of the TME, MSCs regulate GC cell metabolic reprogramming by delivering functional molecules such as nucleic acids and proteins via exosomes. MSCs are centrally involved in chemoresistance, proliferation, metastasis, and stemness maintenance, forming a bidirectional regulatory loop with tumor cells that serves as a vital node connecting stromal cells to tumor metabolism within the metabolic-immune axis. The mechanisms of exosome-mediated metabolic regulation are diverse and closely linked to signaling pathway activation and targeted molecular delivery, providing a key perspective for analyzing metabolic adaptation and therapeutic resistance in GC.

MSC-derived exosomes drive GC chemoresistance by delivering non-coding RNAs that target metabolism-related genes and signaling pathways. For instance, miR-301b-3p in MSC exosomes directly targets thioredoxin-interacting protein (TXNIP), upregulating the expression of multidrug resistance-associated proteins (e.g., P-gp, MRP) to enhance resistance to cisplatin and vincristine while inhibiting apoptosis (79). Exosomes from GC tissue-derived MSCs transfer upregulated miR-221 to GC cells, promoting proliferation and migration through paracrine effects; its expression levels are closely correlated with lymph node metastasis and TNM staging (80). Furthermore, hypoxia induces high expression of lncRNA HIF1A-AS3 in MSCs, which regulates the miR-142-3p/miR-24-3p/PROX1 axis via exosome-mediated siRNA delivery to activate the β-catenin pathway. This induces the transition of MSCs into CAFs, indirectly strengthening GC cell resistance to oxaliplatin (110).

Protein molecules and signaling pathway activation represent another core mechanism by which MSC exosomes regulate GC metabolism. Exosomes from p53-deficient MSCs are enriched with the ubiquitin ligase UBR2, which, upon delivery to GC cells, activates the Wnt/β-catenin pathway and upregulates downstream genes such as CD44 and CyclinD1 to promote tumor growth, metastasis, and stemness maintenance (61). MSC exosomes can also activate the CaM-Ks/Raf/MEK/ERK pathway to antagonize 5-fluorouracil-induced apoptosis and increase the expression of resistance-related proteins, directly driving metabolic adaptation and chemoresistance (111). Simultaneously, activation of the AKT pathway induces EMT and upregulates stemness markers like Oct4 and Lin28B, reinforcing the malignant phenotype and metabolic plasticity of GC cells (112). Additionally, human bone marrow MSC (BM-MSC) exosomes activate the Hedgehog pathway to promote GC cell growth; inhibiting this pathway can reverse these pro-tumor metabolic effects (113).

A bidirectional metabolic regulatory loop exists between MSCs and GC cells, achieving signal feedback and phenotypic remodeling through exosomes. Lymph node metastasis-derived GC cells secrete Wnt5a-rich exosomes that activate the YAP signaling pathway in BM-MSCs, achieving specific reprogramming that incorporates MSCs into the metastatic microenvironment to maintain a pro-tumor phenotype (62). Exosomes from the AGS cell line activate the NF-κB pathway in MSCs, enhancing their ability to activate immune cells and maintaining an inflammatory microenvironment that indirectly feeds back into tumor metabolism and growth (114). Furthermore, MSC exosomes can be modified to improve metabolic targeting; for example, human umbilical cord MSC small extracellular vesicles modified by neutrophil membrane fusion can enrich PTX3 protein, extending circulation time and enhancing inhibitory effects on GC metabolism (115). MSC exosome delivery of L-PGDS can inhibit STAT3 phosphorylation and downregulate stemness markers, thereby blocking GC metabolic reprogramming (64).

In summary, MSCs participate in GC metabolic reprogramming across multiple dimensions—from direct metabolic regulation and induction of resistance to indirect stromal phenotype remodeling and the formation of bidirectional loops. This mechanism deepens our understanding of the synergy between the GC microenvironment and metabolism, providing a critical foundation for developing targeted therapies focused on MSC-derived exosomes.

#### 5.2.4 Lymphocytes

As the core effectors of both adaptive and innate immunity, lymphocytes—comprising key subpopulations such as T cells and natural killer (NK) cells—serve as central targets in the reciprocal regulation between immune function and tumor metabolism within the metabolic-immune axis (116). GC-derived exosomes multi-dimensionally impair anti-tumor functions of lymphocytes by delivering functional cargoes such as PD-L1 and non-coding RNAs, ranging from direct inhibition of activity and induction of functional exhaustion to the indirect remodeling of the immunosuppressive microenvironment. Simultaneously, feedback via metabolic signals reinforces tumor metabolic adaptation, providing critical support for immune escape and therapeutic resistance through subpopulation-specific and network-synergistic mechanisms (54, 99, 117).

T cells are the primary targets of exosomal regulation, where exosomes induce functional exhaustion associated with metabolic reprogramming through dual mechanisms. On one hand, PD-L1 enriched on the exosomal surface can directly bind to PD-1 on CD8^+^ T cells, inhibiting their proliferation, killing capacity, and secretion of cytokines (e.g., IFN-γ, TNF-α), thereby directly blocking effector immunity (92). Histone demethylase 1 (LSD1) can induce the accumulation of exosomal PD-L1 in GC cells, further suppressing T-cell responses; conversely, LSD1 knockdown reduces exosomal PD-L1 and restores T-cell function (54). On the other hand, exosomal nucleic acids regulate T-cell transcription and metabolism: circ_0001947 upregulates CD39 expression by sponging miR-661/miR-671-5p, driving CD8^+^ T-cell exhaustion and resistance to anti-PD-1 therapy (45). Additionally, circMAN1A2 binds to the SFPQ protein to inhibit T-cell receptor signaling pathway activation (118), while miR-135b-5p targets the SP1 gene to decrease the survival rate of Vγ9Vδ2 T cells and reduce cytotoxic cytokine production (119).

NK cells, as the core effectors of innate immunity, are also precisely regulated by exosomes. Highly expressed miR-552-5p in the plasma exosomes of GC patients downregulates activation receptors such as NKG2D and NKp30 on NK cells, inhibiting the secretion of perforin, granzymes, and IFN-γ, thus weakening their cytotoxicity (81).

Regulatory T cells (Tregs) serve as key mediators in exosome-constructed immunosuppressive networks, indirectly feeding back into tumor metabolism. GC-derived exosomes rich in TGF-β1 can induce initial CD4^+^ T cells to express Treg characteristic molecules such as FOXP3 and CTLA-4, increasing the local Treg ratio and deepening immunosuppression (63). Simultaneously, exosomes indirectly promote Treg function by remodeling stromal cells: GC EVs activate the TGF-β/STAT5 pathway in fibroblasts, upregulating FOXP3 expression and the secretion of inhibitory cytokines like IL-10, which restricts CD8^+^ T-cell infiltration and reinforces the immunosuppressive barrier (120). Moreover, lncRNA RP11-323N12.5 drives Treg differentiation via the c-Myc/YAP1 axis, forming a positive feedback loop between immunosuppression and tumor growth (121).

The mechanisms by which exosomes regulate lymphocytes offer diverse directions for clinical translation. Low levels of circulating exosomal lncRNA-GC1 are associated with increased activated CD8^+^ T cells and favorable immunotherapy outcomes, serving as a potential predictive biomarker (122).

In conclusion, GC-derived exosomes construct specific regulatory networks that influence multiple lymphocyte subpopulations. By directly inhibiting effector functions, inducing exhaustion, and indirectly strengthening Treg-mediated immunosuppression, they create a synergistic effect between immune dysfunction and metabolic adaptation, representing a core mechanism of tumor immune escape and therapeutic resistance.

#### 5.2.5 Cellular heterogeneity shapes exosome-mediated metabolic–immune communication

Beyond the functions of CAFs, TAMs, MSCs, and lymphocytes described above, the heterogeneity of these EV-producing populations represents an important factor influencing EV-mediated communication. Recent single-cell and spatial studies have demonstrated that these cell populations comprise multiple functional states with distinct metabolic programs, signaling activities, and secretory profiles (123–125). Therefore, exosomes released from different cellular states may contain distinct molecular cargoes and exert divergent biological effects.

CAFs provide an illustrative example. GC-associated fibroblasts include inflammatory CAFs (iCAFs), myofibroblastic CAFs (myCAFs), and antigen-presenting CAFs (apCAFs), which differ in their spatial distribution and interactions with tumor cells (125). Although these findings suggest that CAF-derived exosomes may be functionally heterogeneous, subtype-resolved characterization of CAF EV cargo and recipient-cell effects in GC remains limited.

Similarly, TAMs represent a continuum of transcriptional and functional states rather than a simple M1/M2 classification (123, 124). Different macrophage states may generate exosomes with distinct immunoregulatory and metabolic functions, thereby influencing tumor metabolism, immune suppression, angiogenesis, and therapy resistance. MSC-derived exosomes are also affected by tissue origin and culture conditions, which may contribute to variations in their molecular composition and biological activity (126).

However, direct evidence linking specific cellular states to defined EV cargo and functional outcomes remains scarce in GC. Most current studies rely on bulk cell populations or conventional cell lines, making it difficult to determine which cellular subtype generates specific EV-mediated effects. Future studies integrating single-cell/spatial profiling with EV transcriptomics, proteomics, and metabolomics will be required to define subtype-specific EV communication networks. Key questions include whether distinct CAF, TAM, or MSC states selectively package metabolic regulators into exosomes and whether these EV populations exhibit different recipient-cell preferences during GC progression and treatment resistance.

GC comprises clinically and molecularly distinct diseases, including intestinal- and diffuse-type tumors and the EBV-positive, microsatellite-unstable, chromosomal-instability, and genomically stable subtypes (127, 128). However, most GC extracellular-vesicle studies have not stratified samples according to these classifications. Moreover, vesicles isolated from established cell lines, patient-derived organoids, tumor tissues, plasma, serum, or malignant ascites represent biologically and technically distinct sources, and their cargo profiles should not be considered interchangeable (129). Future studies should integrate molecular-subtype annotation with matched tissue- and biofluid-derived EV profiling to determine whether subtype-specific vesicle signatures have reproducible biological and clinical significance.

## 6 Diagnostic and therapeutic strategies targeting the metabolic-immune axis

**Figure 4:**
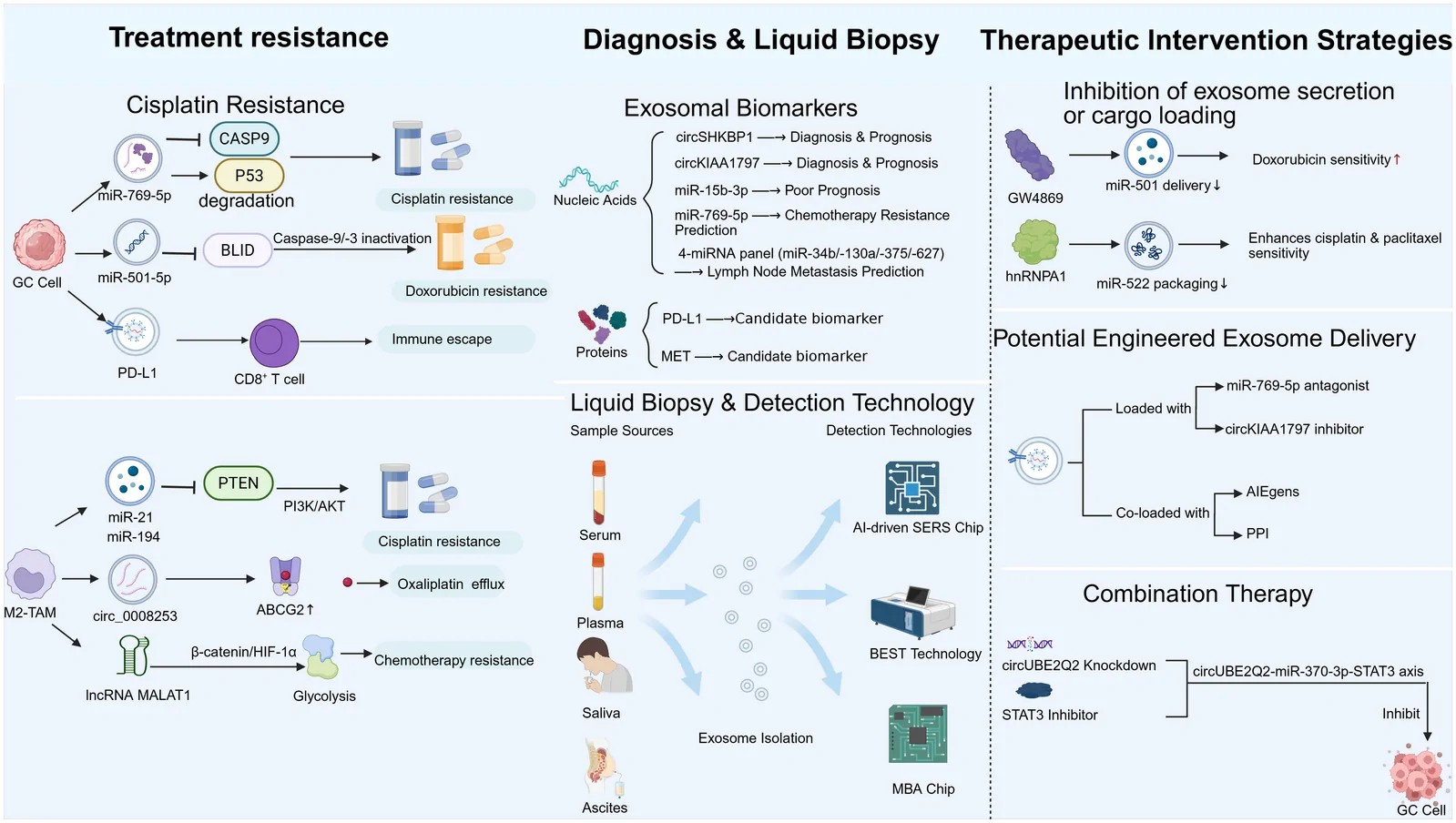
Exosome-mediated treatment resistance, liquid-biopsy applications, and therapeutic strategies in gastric cancer. The left panel summarizes representative resistance mechanisms. Resistant-GC-cell-derived exosomal miR-769-5p suppresses CASP9 and promotes p53 degradation, whereas miR-501-5p suppresses BLID and inactivates caspase-9/-3 signaling. M2-like-TAM-derived miR-21 and miR-194 suppress PTEN and activate PI3K/AKT signaling. Exosomal circ_0008253 promotes oxaliplatin efflux through ABCG2, while MALAT1 enhances glycolytic adaptation through β-catenin/HIF-1α signaling. Exosomal PD-L1 suppresses CD8^+^ T-cell function and may contribute to immune escape and resistance to immune-checkpoint blockade. The middle panel illustrates candidate exosomal nucleic-acid and protein biomarkers detectable in serum, plasma, saliva, or ascites, together with emerging isolation and detection platforms. These biomarkers remain at different stages of clinical validation. The right panel presents therapeutic approaches, including GW4869-mediated inhibition of exosome release, disruption of hnRNPA1-dependent miR-522 loading, experimental engineered-exosome delivery, and combination targeting of the circUBE2Q2–miR-370-3p–STAT3 axis. Candidate engineered-exosome interventions should be considered preclinical or conceptual unless specifically supported by *in vivo* GC experiments. AIEgen, aggregation-induced emission luminogen; GC, gastric cancer; MBA, multiparameter biochip assay; PPI, proton-pump inhibitor; SERS, surface-enhanced Raman scattering; TAM, tumor-associated macrophage.

Based on the core role of the exosome-mediated metabolic-immune regulatory axis in tumor progression and resistance, developing diagnostic biomarkers and therapeutic intervention strategies targeting key nodes of this axis has become a vital direction for precision medicine in oncology. Combining strategies such as liquid biopsy, targeted exosomal communication, and combined metabolic and immune targeting is expected to break through existing therapeutic bottlenecks and improve patient prognosis (**Figure 4**).

### 6.1 Diagnostic and prognostic biomarkers (liquid biopsy applications)

As core vehicles for liquid biopsy, the nucleic acids, proteins, and other molecules carried by exosomes serve as ideal biomarkers for early tumor diagnosis, prognostic assessment, and prediction of treatment response. In GC, serum levels of exosomal circKIAA1797 and circSHKBP1 are significantly elevated and correlate with TNM staging and survival rates. Specifically, exosomal circSHKBP1 levels significantly decrease after gastrectomy, making it a viable marker for GC diagnosis and prognostic evaluation (42, 84). Additionally, an exosomal miRNA panel composed of miR-34b, miR-130a, miR-375, and miR-627 demonstrated high predictive performance for lymph node metastasis in high-risk early GC patients (AUC 0.86), with a clinical risk factor-integrated stratification model achieving an AUC of 0.94 (130).

Furthermore, high expression of serum exosomal miR-15b-3p in GC patients is associated with poor prognosis (AUC 0.820) (131). In cisplatin-resistant GC patients, the enrichment of miR-769-5p in serum exosomes serves as a predictive biomarker for chemoresistance (68). In the field of pan-cancer diagnosis, an AI-driven SERS chip based on exosomes can accurately distinguish between ten common cancers, including GC, by detecting serum exosomal molecular fingerprints with an accuracy of 93.89%. Within this system, exosomal deoxyadenosine triphosphate has emerged as a potential pan-cancer biomarker (132). Moreover, the combination of Bessel beam excitation stimulated separation technology (BEST) and multi-parameter biochip assay (MBA) allows for the detection of exosomal miR-140-5p and miR-301a-3p in plasma and saliva, with salivary exosomal markers showing superior diagnostic efficacy compared to those in plasma and tissue (133).

### 6.2 Therapeutic intervention strategies

#### 6.2.1 Targeting exosomal communication

Inhibition of exosome biogenesis, secretion, or uptake to block the transmission of mediated metabolic-immune regulatory signals represents an effective strategy for reversing tumor resistance. In GC, pretreatment with GW4869 (an exosome secretion inhibitor) can suppress the secretion of exosomal miR-501 from doxorubicin-resistant GC cells, thereby restoring the sensitivity of previously resistant cells to doxorubicin (69). Furthermore, inhibiting the ubiquitination-mediated stabilization of hnRNPA1 in CAFs reduces the loading and secretion of exosomal miR-522, which enhances the chemosensitivity of GC cells to cisplatin and paclitaxel (32). Furthermore, surface engineering or receptor-directed interception may offer future approaches for modifying EV uptake. However, GC-specific evidence demonstrating selective blockade of EV uptake by immune or stromal cells remains limited (134).

#### 6.2.2 Combined targeting of metabolic and immune checkpoints

Synergistic therapeutic effects can be achieved by simultaneously targeting key nodes of the exosome-mediated metabolic-immune regulatory axis, such as metabolic enzymes, signaling pathways, and immune checkpoints. In GC, modified Jianpi Yangzheng (mJPYZ) induces tumor cell apoptosis by targeting PKM2 to downregulate the PI3K/Akt/mTOR pathway. Simultaneously, it reduces the abundance of serum exosomal PKM2, thereby decreasing the induction of M2-TAM polarization and inhibiting tumor progression (58). Furthermore, the combined treatment of circUBE2Q2 knockdown and STAT3 inhibitors synergistically suppresses GC growth by modulating the circ_UBE2Q2/miR-370-3p/STAT3 axis and disrupting exosomal communication (135).

#### 6.2.3 Engineered exosomes as therapeutic delivery systems

Engineered exosomes have attracted considerable attention as potential therapeutic delivery platforms because of their ability to transport nucleic acids, proteins, and small molecules. However, their therapeutic application should not rely on the assumption that exosomes possess inherent and highly specific targeting capability. Exosome uptake is mediated by multiple mechanisms, including receptor–ligand interactions, endocytosis, phagocytosis, and membrane fusion (136). Therefore, whether GC-derived exosomes preferentially recognize specific tumor or stromal populations *in vivo* remains insufficiently established. Current evidence mainly demonstrates exosome-mediated biological communication rather than selective therapeutic targeting. For example, endogenous exosome-transmitted miR-769-5p from cisplatin-resistant GC cells promotes resistance by suppressing CASP9 and impairing p53-mediated apoptosis (68). This finding identifies miR-769-5p as a potential therapeutic target. Thus, exosome-based interventions targeting resistance-associated cargoes should be considered promising hypotheses rather than established therapeutic approaches.

A representative proof-of-concept study used tumor-derived exosomes to co-deliver aggregation-induced emission luminogens and proton-pump inhibitors, thereby inhibiting glutamine-dependent antioxidant defense, enhancing type-I photodynamic therapy, and promoting immunogenic cell death in an MGC803-derived GC model (137). However, because this evidence was obtained from a single cell-line-derived model, it demonstrates the feasibility of exosome-assisted combination delivery rather than GC-selective targeting or clinical efficacy.

To improve delivery specificity, exosome surfaces can be modified using targeting peptides, antibodies, aptamers, or engineered membrane proteins. Nevertheless, modified exosomes may still undergo uptake by clearance organs, particularly the mononuclear phagocyte system (134). Therefore, future studies should evaluate not only tumor accumulation but also cell-type-specific uptake, biodistribution, pharmacokinetics, and functional cargo delivery.

For GC therapy, orthotopic models, humanized mouse models, and patient-derived organoid systems will be essential to determine whether engineered exosomes can reproduce the complex metabolic and immune interactions observed in human tumors. In particular, multicellular organoid systems incorporating tumor cells, CAFs, and immune cells may provide a more physiologically relevant platform for evaluating exosome-mediated metabolic–immune modulation.

In GC with peritoneal dissemination, engineered exosome delivery faces additional disease-specific barriers. Therapeutic vesicles must distribute across multiple peritoneal tumor nodules, penetrate a fibrotic and ascites-rich microenvironment, and avoid rapid sequestration by peritoneal macrophages and mesothelial surfaces. These challenges should be evaluated in orthotopic and peritoneal-metastasis models rather than inferred solely from subcutaneous xenografts.

Overall, engineered exosomes represent a promising strategy for intervening in the metabolic–immune network of GC, but successful translation requires overcoming unresolved challenges in targeting specificity, biological distribution, manufacturing consistency, and safety evaluation.

#### 6.2.4 Translational limitations of current experimental models

Despite promising mechanistic findings, the clinical translation of exosome-based therapeutic strategies in GC remains challenging because most available evidence originates from established cell lines and cell-line-derived xenograft models. Although these systems have provided valuable insights into exosome-mediated signaling pathways, they cannot fully reproduce the cellular heterogeneity, immune landscape, stromal composition, and metabolic complexity of human GC.

Therefore, key mechanisms identified in experimental models require validation using complementary systems. Patient-derived organoids (PDOs) can preserve important genetic and phenotypic characteristics of individual tumors and enable personalized therapeutic testing, although conventional organoids lack complete immune and stromal components (138, 139). PDX models better maintain tumor architecture but have limitations related to immune deficiency and murine stromal replacement. Humanized mouse and orthotopic models may provide additional information regarding immune interactions, anatomical distribution, and therapeutic responses.

Spatial transcriptomics and other spatial multi-omics approaches can further determine whether experimentally identified exosome-mediated interactions occur within human tumor tissues. Ultimately, integration of patient-derived models, immune-competent systems, and longitudinal clinical samples will be required to establish the translational relevance of the proposed bidirectional metabolic–immune cycle.

## 7 Methodological heterogeneity and hierarchy of evidence

The proposed bidirectional malignant-cycle model should be interpreted in the context of substantial methodological heterogeneity across extracellular-vesicle studies. Differences in vesicle isolation and purification—including ultracentrifugation, size-exclusion chromatography, polymer precipitation, and immunoaffinity capture—as well as differences in sample type, normalization strategy, and characterization criteria can alter the apparent molecular composition and biological activity of isolated vesicle populations. According to the MISEV2023 recommendations, the term “exosome” should be used cautiously when an endosomal origin has not been experimentally demonstrated; consequently, some findings summarized in this review may be more appropriately interpreted as extracellular-vesicle-associated rather than definitively exosome-specific (129).

To distinguish convergent biological evidence from direct replication of individual molecular pathways, the evidence underlying the proposed framework can be divided into two levels. At the process level, three conclusions show convergent support across multiple independent studies, although methodological heterogeneity precludes their classification as high-certainty evidence. First, intercellular vesicle transfer contributes to chemoresistance in GC. This conclusion is supported by studies involving resistant tumor-cell-derived miR-501 (69) and miR-769-5p (68), macrophage- or TAM-derived miR-3681-3p (70), miR-223 (71), miR-21 and miR-194 (72, 73), MSC-derived miR-301b-3p (79), and TAM-derived CRNDE (83), which converge on impaired apoptosis, defective DNA repair, drug-efflux activation, and metabolic adaptation. Second, tumor-derived vesicles contribute to immune suppression through multiple independently reported mechanisms, including PD-L1-related signaling (54), TGF-β1-mediated Treg induction (63), circ_0001947-associated CD8^+^ T-cell exhaustion (45), miR-552-5p-mediated NK-cell dysfunction (81), circMAN1A2-mediated suppression of T-cell activity (118), and miR-135b-5p-mediated impairment of Vγ9Vδ2 T cells (119). Third, bidirectional communication between GC cells and stromal or immune cells contributes to metabolic adaptation and malignant progression. This process-level conclusion is supported by independent observations involving CAF-derived miR-522 (32), tumor-derived PKM2 (56), M2-TAM-derived MALAT1 (82) and CRNDE (83), and tumor-derived circATP8A1 (8), miR-519a-3p (51), let-7g-5p (77), and miR-92b-5p (78), which regulate macrophage state. Collectively, these studies provide convergent support for chemoresistance transfer, immune suppression, and tumor–microenvironment metabolic feedback as reproducible biological processes.

Importantly, high confidence in these process-level conclusions should not be interpreted as independent validation of every individual cargo–target pathway. Most specific molecular mechanisms remain supported by a single primary report or by studies from one research group. Examples include the hypoxic exosomal miR-301a-3p–PHD3–HIF-1α feedback loop (35), CAF-derived exosomal miR-522-mediated inhibition of ALOX15-dependent ferroptosis (32), exosomal circPTBP3-mediated activation of the TFAP2B–SGK1 pathway in peritoneal mesothelial cells (41), ascites-derived exosomal MET signaling (43), the miR-769-5p–CASP9/p53 pathway in cisplatin resistance (68), and the circKIAA1797–miR-4429–PBX3 axis (84). These mechanisms are biologically plausible and often supported by internally consistent loss-of-function, gain-of-function, or rescue experiments; however, until reproduced by independent laboratories using complementary models, they should be regarded as promising mechanistic hypotheses rather than established general principles of GC biology.

Future studies should therefore distinguish conceptual convergence from molecular replication. A cargo–target pathway should be considered strongly validated only when it has been reproduced by at least two independent groups, supported by orthogonal vesicle-characterization approaches, confirmed through cargo-depletion and rescue experiments, and demonstrated across complementary models such as patient-derived organoids, orthotopic or humanized mouse models, and independent clinical cohorts. Standardized reporting under MISEV2023 and EV-TRACK—including detailed documentation of isolation, purification, particle characterization, normalization, contamination assessment, biodistribution, and functional validation—will be essential for determining which exosome-associated mechanisms represent reproducible biological principles and which remain study-specific observations (129, 140).

## 8 Conclusion and perspectives

Over the past decade, exosomes in GC have evolved from passive carriers of cellular materials into active regulators of tumor–microenvironment communication. By integrating evidence from tumor cells, CAFs, TAMs, MSCs, endothelial cells, and lymphocytes, this review proposes a bidirectional malignant-cycle model in which exosome-mediated metabolic reprogramming and immune remodeling continuously reinforce each other.

**Figure 5:**
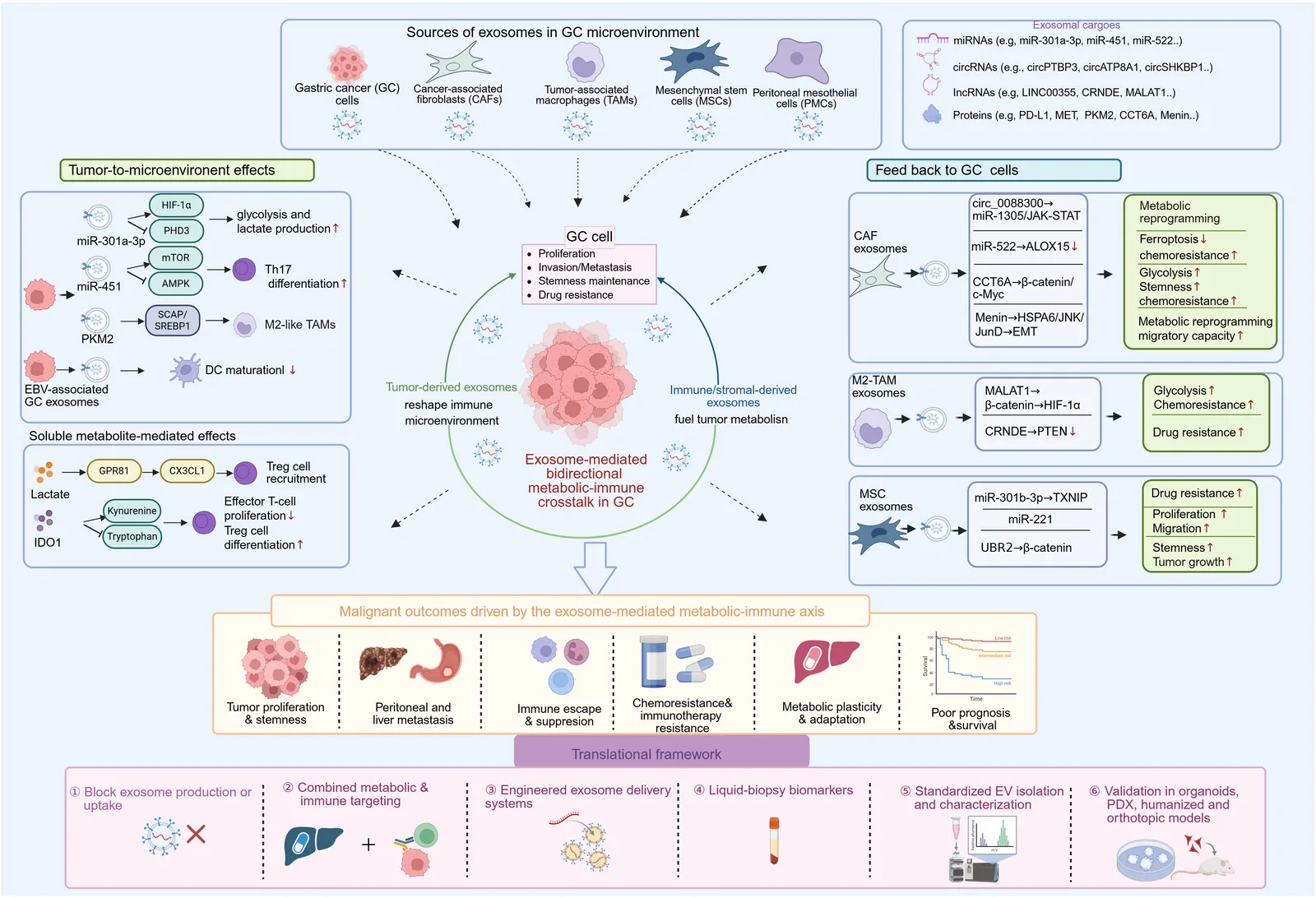
Integrative model and translational framework of the exosome-associated metabolic–immune axis in gastric cancer. GC cells and diverse microenvironmental populations, including CAFs, TAMs, MSCs, and PMCs, release EVs containing distinct nucleic-acid and protein cargoes. Tumor-derived EVs influence immune and stromal cells by modifying glycolytic adaptation, macrophage lipid metabolism, dendritic-cell maturation, and lymphocyte function. Soluble metabolic signals, including lactate and products of IDO1-mediated tryptophan catabolism, operate in parallel with EV-mediated communication and should not be interpreted as established exosomal cargoes in GC. Conversely, CAF-, TAM-, and MSC-derived EVs feed back to GC cells through cargo-dependent regulation of glycolysis, ferroptosis, stemness, migration, and treatment resistance. These reciprocal interactions contribute to tumor proliferation, peritoneal and hepatic metastasis, immune escape, chemoresistance, immunotherapy resistance, metabolic plasticity, and reduced survival. The lower panel summarizes potential translational directions, including inhibition of EV production or uptake, combined metabolic and immune targeting, engineered-exosome delivery, liquid-biopsy biomarker development, standardized EV isolation and characterization, and validation in organoids, PDX models, humanized mice, and orthotopic models. Individual molecular pathways differ substantially in their level of experimental validation, and most remain supported by single primary studies. CAF, cancer-associated fibroblast; EV, extracellular vesicle; GC, gastric cancer; IDO1, indoleamine 2, 3-dioxygenase 1; MSC, mesenchymal stromal cell; PDX, patient-derived xenograft; PMC, peritoneal mesothelial cell; TAM, tumor-associated macrophage.

In this framework, metabolic stressors such as hypoxia, nutrient limitation, altered lipid metabolism, and therapeutic pressure reshape exosome production and cargo composition. These exosomes subsequently reprogram stromal and immune cells, promoting immune suppression, CAF activation, angiogenesis, and treatment resistance. Conversely, exosomes released from reprogrammed stromal and immune cells feed back to tumor cells by enhancing glycolysis, metabolic adaptation, stemness, and survival. This reciprocal communication provides a mechanistic explanation for why metabolic plasticity and immune escape frequently coexist during GC progression (**Figure 5**).

This model also suggests a conceptual shift in therapeutic development. Rather than targeting isolated intracellular pathways, future strategies may need to disrupt critical intercellular feedback nodes within the exosome-mediated network. In chemotherapy, blocking the secretion, packaging, or uptake of resistance-associated exosomal cargoes may restore ferroptosis or apoptosis and thereby enhance treatment sensitivity. In immunotherapy, inhibiting exosome-mediated immunosuppressive and metabolic signals together with immune-checkpoint blockade may improve effector-cell function and antitumor responses. In targeted therapy, microenvironment-derived exosomal feedback should be considered a potential mechanism of adaptive pathway reactivation. For example, ascites-derived exosomal MET can activate oncogenic signaling in recipient GC cells; therefore, whether combining MET inhibition with blockade of exosomal MET transfer or uptake provides greater and more durable pathway suppression than MET inhibition alone represents a testable strategy, particularly in patients with MET-enriched EVs.

The proposed framework generates several testable hypotheses. First, because CAF-derived exosomal miR-522 suppresses ALOX15-dependent lipid peroxidation and ferroptosis, inhibition of exosomal miR-522 may restore ferroptosis sensitivity and enhance the efficacy of chemotherapy. Second, combining inhibition of CAF-derived exosomal miR-522 with anti-PD-1 therapy may simultaneously weaken stromal metabolic protection and reinvigorate antitumor T-cell activity, thereby overcoming resistance more effectively than either intervention alone. This hypothesis should be tested initially in immunocompetent or humanized GC models that preserve CAF–tumor–immune interactions. Third, longitudinal profiling of circulating exosomal cargoes during chemotherapy, immune-checkpoint blockade, or targeted therapy may determine whether alterations in miR-522, PD-L1, MET, or other resistance-associated molecules precede clinical progression and could therefore guide early combination intervention.

However, several challenges remain. First, exosome-producing populations such as CAFs, TAMs, and MSCs are highly heterogeneous, and subtype-specific exosome functions remain poorly defined. Second, exosome uptake involves both specific and non-specific mechanisms, and selective targeting of recipient cells *in vivo* has not been systematically demonstrated. Third, most mechanistic evidence derives from simplified experimental models that cannot fully represent human GC complexity. Finally, methodological differences in exosome isolation and characterization limit cross-study comparability.

Addressing these challenges will require integration of single-cell and spatial multi-omics, standardized exosome characterization, patient-derived models, and clinically annotated longitudinal cohorts. Ultimately, the therapeutic potential of exosomes will depend not only on identifying individual cargo molecules but also on understanding when and how exosome-mediated metabolic–immune feedback becomes therapeutically actionable.

## References

[1] Sung H Filho AM Laversanne M Ferlay J Siegel RL Soerjomataram I . Global cancer statistics 2024: Globocan estimates of incidence and mortality worldwide for 34 cancers in 186 countries. CA: A Cancer J For Clin. (2026) 76:e70090. doi: 10.3322/caac.70090 42417444 PMC13343830

[2] Sun Y Nie W Xiahou Z Wang X Liu W Liu Z . Integrative single-cell and spatial transcriptomics uncover Elk4-mediated mechanisms in Ndufab1+ tumor cells driving gastric cancer progression, metabolic reprogramming, and immune evasion. Front Immunol. (2025) 16:1591123. doi: 10.3389/fimmu.2025.1591123 40688093 PMC12271198

[3] Sun Q Li S Lou J Wang X Xu X . Recent advances in tumour microenvironment impact immunotherapy resistance in gastric cancer. Crit Rev Oncol/Hematol. (2025) 215:104837. doi: 10.1016/j.critrevonc.2025.104837 40618861

[4] Gui F Zhang L Xiao J Zeng C . Decoding the role of intratumoral microbiota in gastric cancer. Biochim Biophys Acta Rev Cancer. (2025) 1880:189355. doi: 10.1016/j.bbcan.2025.189355 40409517

[5] Chen J Zhang Y Zhang T Wen Z Li Y Ye Y . Research progress on chronic stress stimulation and gastric cancer metastasis. Disc Oncol. (2025) 16:2160. doi: 10.1007/s12672-025-03950-w 41288846 PMC12647482

[6] Yu X Wang L Zhu L . Intraperitoneal metastasis of gastric cancer: New insights on resident macrophages. Int J Surg. (2026) 112(2):4547–62. doi: 10.1097/JS9.0000000000003944 41233166

[7] Li Q Zhu CC Ni B Zhang ZZ Jiang SH Hu LP . Lysyl oxidase promotes liver metastasis of gastric cancer via facilitating the reciprocal interactions between tumor cells and cancer associated fibroblasts. EBioMedicine. (2019) 49:157–71. doi: 10.1016/j.ebiom.2019.10.037 31678002 PMC7113184

[8] Deng C Huo M Chu H Zhuang X Deng G Li W . Exosome circATP8A1 induces macrophage M2 polarization by regulating the miR-1-3p/STAT6 axis to promote gastric cancer progression. Mol Cancer. (2024) 23:49. doi: 10.1186/s12943-024-01966-4 38459596 PMC10921793

[9] Liang Y Liu Y Zhang Q Zhang H Du J . Tumor-derived extracellular vesicles containing microrna-1290 promote immune escape of cancer cells through the grhl2/zeb1/pd-l1 axis in gastric cancer. Trans Research: J Lab Clin Med. (2021) 231:102–12. doi: 10.1016/j.trsl.2020.12.003 33321257

[10] Zhang Y Cao J Yuan Z Zhou J Zuo H Miao X . Knockdown of SLC7A5 inhibits malignant progression and attenuates oxaliplatin resistance in gastric cancer by suppressing glycolysis. Mol Med (Cambridge Mass). (2025) 31:115. doi: 10.1186/s10020-025-01175-9 40133832 PMC11938572

[11] He Z Lian Z Wu J Xu S Liu S Pan H . PFKFB3 confers cisplatin resistance in gastric cancer by inhibiting ferroptosis through SLC7A11/xCT dephosphorylation. Int Immunopharmacol. (2025) 159:114914. doi: 10.1016/j.intimp.2025.114914 40435821

[12] Wu Y Jiang X Qing H Hou Y Ma Y Wang T . Targeting the Anxa8-Sp1-Ppa1 axis to modulate TCA cycle and matrix deposition in diffuse-type gastric cancer. Res (Washington DC). (2025) 8:838. doi: 10.34133/research.0838 40862051 PMC12377485

[13] Won Y Jang B Lee SH Reyzer ML Presentation KS Kim H . Oncogenic fatty acid metabolism rewires energy supply chain in gastric carcinogenesis. Gastroenterology. (2024) 166:772–786.e14. doi: 10.1053/j.gastro.2024.01.027 38272100 PMC11040571

[14] Peng C Zhang F Zhou F Tan S Xie W . Lncrna Hcp5: A key regulator of tumor metabolic reprogramming, signaling pathway modulation, and therapeutic resistance. Int J Biol Macromol. (2025) 333:148606. doi: 10.1016/j.ijbiomac.2025.148606 41161440

[15] Zhang C Chong X Jiang F Gao J Chen Y Jia K . Plasma extracellular vesicle derived protein profile predicting and monitoring immunotherapeutic outcomes of gastric cancer. J Extracell Vesicles. (2022) 11:e12209. doi: 10.1002/jev2.12209 35362262 PMC8971562

[16] Wang Q Sun Y Li J Li Z Yuan F Xia Z . Targeting Linc01711 in Fap(+) cancer-associated fibroblasts overcomes lactate-mediated immunosuppression and enhances anti-Pd-1 efficacy in lung adenocarcinoma. Cell Death Dis. (2025) 16:642. doi: 10.1038/s41419-025-07974-6 40855046 PMC12379239

[17] Chai H Hu Q Yao S Ma S Su W . Endoplasmic reticulum stress-mediated programmed cell death in the tumor microenvironment. Cell Death Discov. (2025) 11:559. doi: 10.1038/s41420-025-02862-6 41407677 PMC12712068

[18] Ding X Zhou J Wang Z Bo Y Abliz Z An Z . Lipidomic machine learning predictor for progression of gastric cancer. Clinica Chimica Acta; Int J Clin Chem. (2026) 580:120731. doi: 10.1016/j.cca.2025.120731 41274332

[19] Cao R Zhou F Zhu C Xu H . Metabolic reprogramming as a key regulator in Helicobacter pylori-infected gastric cancer. Gastric Cancer. (2026) 29:1–15. doi: 10.1007/s10120-025-01675-x 41171531 PMC12830420

[20] Wu Z Wang H Zheng Z Lin Y Bian L Geng H . Ido1 inhibition enhances Cldn18.2-CAR-T cell therapy in gastrointestinal cancers by overcoming kynurenine-mediated metabolic suppression in the tumor microenvironment. J Transl Med. (2025) 23:275. doi: 10.1186/s12967-025-06276-x 40045363 PMC11884131

[21] Liang P Li Z Chen Z Chen Z Jin T He F . Metabolic reprogramming of glycolysis, lipids, and amino acids in tumors: Impact on Cd8+ T cell function and targeted therapeutic strategies. FASEB Journ: Off Publ Fed Am Soc For Exp Biol. (2025) 39:e70520. doi: 10.1096/fj.202403019R 40249661

[22] Chiarugi P Cirri P . Metabolic exchanges within tumor microenvironment. Cancer Lett. (2016) 380:272–80. doi: 10.1016/j.canlet.2015.10.027 26546872

[23] Moiseeva E Sergeev I Chernyshev V Zaborova O Kohzevnikova D Yakovlev A . Profiling of Cd63 and Epcam membrane proteins of extracellular vesicles on tannic acid-coated magnetic beads using conventional flow cytometry. Int J Mol Sci. (2025) 26(23):11324. doi: 10.3390/ijms262311324 41373484 PMC12692446

[24] Panda SS Sahoo RK Patra SK Behera S Khamari AK Biswal BK . Exosomal heterogeneity and functional zonation in cancer drug resistance. Drug Discov Today. (2025) 31:104587. doi: 10.1016/j.drudis.2025.104587 41418930

[25] Yi YW Lee JH Kim SY Pack CG Ha DH Park SR . Advances in analysis of biodistribution of exosomes by molecular imaging. Int J Mol Sci. (2020) 21(2):665. doi: 10.3390/ijms21020665 31963931 PMC7014306

[26] Pan Y Zhang B Li Z Hu K . Extracellular vesicles-associated trna-derived fragments: Emerging insights into cancer progression and clinical application potential. Genes Dis. (2026) 13:101682. doi: 10.1016/j.gendis.2025.101682 41376858 PMC12688670

[27] Shen H Li S Lin L Wu Q Dong Z Xu W . Advancements in plant-derived exosome-like vesicles: Versatile bioactive carriers for targeted drug delivery systems. J Pharm Anal. (2025) 15:101300. doi: 10.1016/j.jpha.2025.101300 41450858 PMC12731909

[28] Fu X Ruan X He J . Mettl3-driven m^6^a epigenetics in gastric cancer: Unveiling oncogenic networks and clinical translation from tumorigenesis to therapy resistance. Cell Biol Toxicol. (2025) 41:132. doi: 10.1007/s10565-025-10086-8 41003789 PMC12474612

[29] Li S Zhou J Wang S Yang Q Nie S Ji C . N(6)-methyladenosine-regulated exosome biogenesis orchestrates an immunosuppressive pre-metastatic niche in gastric cancer peritoneal metastasis. Cancer Commun (London England). (2025) 45:941–65. doi: 10.1002/cac2.70034 40370322 PMC12365546

[30] Chen X Song X Li J Wang J Yan Y Yang F . Integrated proteomic, phosphoproteomic, and N-glycoproteomic analyses of small extracellular vesicles from C2c12 myoblasts identify specific PTM patterns in ligand-receptor interactions. Cell Comm Signaling: CCS. (2024) 22:273. doi: 10.1186/s12964-024-01640-8 38755675 PMC11097525

[31] Zhang C Wei G Zhu X Chen X Ma X Hu P . Exosome-delivered circstau2 inhibits the progression of gastric cancer by targeting the mir-589/capza1 axis. Int J Nanomed. (2023) 18:127–42. doi: 10.2147/ijn.S391872 36643863 PMC9832994

[32] Zhang H Deng T Liu R Ning T Yang H Liu D . Caf secreted mir-522 suppresses ferroptosis and promotes acquired chemo-resistance in gastric cancer. Mol Cancer. (2020) 19:43. doi: 10.1186/s12943-020-01168-8 32106859 PMC7045485

[33] Yáñez-Mó M Siljander PR Andreu Z Zavec AB Borràs FE Buzas EI . Biological properties of extracellular vesicles and their physiological functions. J Extracell Vesicles. (2015) 4:27066. doi: 10.3402/jev.v4.27066 25979354 PMC4433489

[34] Gurung S Perocheau D Touramanidou L Baruteau J . The exosome journey: From biogenesis to uptake and intracellular signalling. Cell Comm Signaling: CCS. (2021) 19:47. doi: 10.1186/s12964-021-00730-1 33892745 PMC8063428

[35] Xia X Wang S Ni B Xing S Cao H Zhang Z . Hypoxic gastric cancer-derived exosomes promote progression and metastasis via mir-301a-3p/phd3/hif-1α positive feedback loop. Oncogene. (2020) 39:6231–44. doi: 10.1038/s41388-020-01425-6 32826951

[36] Li Q Li B Li Q Wei S He Z Huang X . Exosomal mir-21-5p derived from gastric cancer promotes peritoneal metastasis via mesothelial-to-mesenchymal transition. Cell Death Dis. (2018) 9:854. doi: 10.1038/s41419-018-0928-8 30154401 PMC6113299

[37] Li P Luo X Xie Y Li P Hu F Chu J . Gc-derived evs enriched with microrna-675-3p contribute to the mapk/pd-l1-mediated tumor immune escape by targeting cxxc4. Mol Ther Nucleic Acids. (2020) 22:615–26. doi: 10.1016/j.omtn.2020.08.020 33230461 PMC7578556

[38] Zhao W Zhang Y Zhang W Sun Y Zheng B Wang J . Exosomal linc00355 promotes the Malignant progression of gastric cancer through histone deacetylase hdac3-mediated tp53inp1 transcriptional inhibition. Life Sci. (2023) 315:121387. doi: 10.1016/j.lfs.2023.121387 36640904

[39] Wang Y Jiang R Zhao H Li F Li Y Zhu M . Ttn-as1 delivered by gastric cancer cell-derived exosome induces gastric cancer progression through *in vivo* and *in vitro* studies. Cell Biol Toxicol. (2023) 39:557–71. doi: 10.1007/s10565-022-09762-w 36214926

[40] Jin G Zhang J Cao T Chen B Tian Y Shi Y . Exosome-mediated lncrna snd1-it1 from gastric cancer cells enhances Malignant transformation of gastric mucosa cells via up-regulating snail1. J Transl Med. (2022) 20:284. doi: 10.1186/s12967-022-03306-w 35739527 PMC9229915

[41] Dong C Zhou Y Shen X Hu S Duan K Chen T . Tumor exosomal circptbp3 drives gastric cancer peritoneal metastasis via mesothelial-mesenchymal transition. Cell Death Dis. (2025) 16:444. doi: 10.1038/s41419-025-07749-z 40500263 PMC12159144

[42] Xie M Yu T Jing X Ma L Fan Y Yang F . Exosomal circshkbp1 promotes gastric cancer progression via regulating the mir-582-3p/hur/vegf axis and suppressing hsp90 degradation. Mol Cancer. (2020) 19:112. doi: 10.1186/s12943-020-01208-3 32600329 PMC7322843

[43] Hyung S Ko J Heo YJ Blum SM Kim ST Park SH . Patient-derived exosomes facilitate therapeutic targeting of oncogenic Met in advanced gastric cancer. Sci Adv. (2023) 9:eadk1098. doi: 10.1126/sciadv.adk1098 38000030 PMC10672184

[44] Fu H Yang H Zhang X Wang B Mao J Li X . Exosomal Trim3 is a novel marker and therapy target for gastric cancer. J Exp Clin Cancer Research: CR. (2018) 37:162. doi: 10.1186/s13046-018-0825-0 30031392 PMC6054744

[45] Wang B Liu W Zhang M Li Y Tang H Wang Y . Circ_0001947 encapsulated by small extracellular vesicles promotes gastric cancer progression and anti-Pd-1 resistance by modulating Cd8(+) T cell exhaustion. J Nanobiotechnol. (2024) 22:563. doi: 10.1186/s12951-024-02826-5 39272146 PMC11401313

[46] Zhao H Yang L Baddour J Achreja A Bernard V Moss T . Tumor microenvironment derived exosomes pleiotropically modulate cancer cell metabolism. eLife. (2016) 5:e10250. doi: 10.7554/eLife.10250 26920219 PMC4841778

[47] Zhu J Wang H Liu Y Li W Cao L Wang W . Exosome-mediated induction of apoptosis in cisplatin-treated gastric cancer cells as a strategy to mitigate side effects. Curr Cancer Drug Targets. (2026) 26(7):855–70. doi: 10.2174/0115680096369817250407164352 40357779

[48] Guo Z Zhang Y Xu W Zhang X Jiang J . Engineered exosome-mediated delivery of circdido1 inhibits gastric cancer progression via regulation of mir-1307-3p/socs2 axis. J Transl Med. (2022) 20:326. doi: 10.1186/s12967-022-03527-z 35864511 PMC9306104

[49] Huang J Wang X Wen J Zhao X Wu C Wang L . Gastric cancer cell-originated small extracellular vesicle induces metabolic reprogramming of Bm-Mscs through Erk-Pparγ-Cpt1a signaling to potentiate lymphatic metastasis. Cancer Cell Int. (2023) 23:87. doi: 10.1186/s12935-023-02935-5 37158903 PMC10169337

[50] Zhang H Deng T Liu R Bai M Zhou L Wang X . Exosome-delivered Egfr regulates liver microenvironment to promote gastric cancer liver metastasis. Nat Commun. (2017) 8:15016. doi: 10.1038/ncomms15016 28393839 PMC5394240

[51] Qiu S Xie L Lu C Gu C Xia Y Lv J . Gastric cancer-derived exosomal mir-519a-3p promotes liver metastasis by inducing intrahepatic m2-like macrophage-mediated angiogenesis. J Exp Clin Cancer Res CR. (2022) 41:296. doi: 10.1186/s13046-022-02499-8 36217165 PMC9549645

[52] Sugimoto A Okuno T Miki Y Tsujio G Sera T Yamamoto Y . Emmprin in extracellular vesicles from peritoneal mesothelial cells stimulates the invasion activity of diffuse-type gastric cancer cells. Cancer Lett. (2021) 521:169–77. doi: 10.1016/j.canlet.2021.08.031 34474145

[53] Wang J Deng R Chen S Deng S Hu Q Xu B . Helicobacter pylori caga promotes immune evasion of gastric cancer by upregulating pd-l1 level in exosomes. iScience. (2023) 26:108414. doi: 10.1016/j.isci.2023.108414 38047083 PMC10692710

[54] Shen DD Pang JR Bi YP Zhao LF Li YR Zhao LJ . Lsd1 deletion decreases exosomal pd-l1 and restores t-cell response in gastric cancer. Mol Cancer. (2022) 21:75. doi: 10.1186/s12943-022-01557-1 35296335 PMC8925194

[55] Xue X Huang J Yu K Chen X He Y Qi D . Yb-1 transferred by gastric cancer exosomes promotes angiogenesis via enhancing the expression of angiogenic factors in vascular endothelial cells. BMC Cancer. (2020) 20:996. doi: 10.1186/s12885-020-07509-6 33054752 PMC7557103

[56] Yuan M Zheng X Zheng S Li H Zhang X Chen Y . Exosomal pkm2: a noninvasive diagnostic marker linking macrophage metabolic reprogramming to gastric cancer pathogenesis. Cancer Sci. (2025) 116:1537–49. doi: 10.1111/cas.70056 40126044 PMC12127107

[57] Gu J Li X Zhao L Yang Y Xue C Gao Y . The role of pkm2 nuclear translocation in the constant activation of the nf-κb signaling pathway in cancer-associated fibroblasts. Cell Death Dis. (2021) 12:291. doi: 10.1038/s41419-021-03579-x 33731686 PMC7969736

[58] Wu J Yuan M Shen J Chen Y Zhang R Chen X . Effect of modified jianpi yangzheng on regulating content of pkm2 in gastric cancer cells-derived exosomes. Phytomed Int J Phytotherapy Phytopharmacol. (2022) 103:154229. doi: 10.1016/j.phymed.2022.154229 35691076

[59] Sun H Zhang T Zhang X Liu Y Wang X Wang X . Exosomal cct6a secreted by cancer-associated fibroblasts interacts with β-catenin to enhance chemoresistance and tumorigenesis in gastric cancer. Advanced Sci (Weinheim Baden-Wurttemberg Germany). (2025) 12:e06674. doi: 10.1002/advs.202506674 40801472 PMC12520511

[60] Wang SH Jiang JX Zheng KT Zhang SJ Chen TD Wang Z . Exosome-derived menin from cancer-associated fibroblasts promotes gastric cancer progression by activating the hspa6/jnk/jund pathway and inducing emt. J Transl Med. (2025) 24(1):274. doi: 10.1186/s12967-025-07539-3 41353438 PMC12918078

[61] Mao J Liang Z Zhang B Yang H Li X Fu H . Ubr2 enriched in p53 deficient mouse bone marrow mesenchymal stem cell-exosome promoted gastric cancer progression via wnt/β-catenin pathway. Stem Cells (Dayton Ohio). (2017) 35:2267–79. doi: 10.1002/stem.2702 28895255

[62] Wang M Zhao X Qiu R Gong Z Huang F Yu W . Lymph node metastasis-derived gastric cancer cells educate bone marrow-derived mesenchymal stem cells via yap signaling activation by exosomal wnt5a. Oncogene. (2021) 40:2296–308. doi: 10.1038/s41388-021-01722-8 33654199 PMC7994201

[63] Yen EY Miaw SC Yu JS Lai IR . Exosomal tgf-β1 is correlated with lymphatic metastasis of gastric cancers. Am J Cancer Res. (2017) 7:2199–208. PMC5714749 29218244

[64] You B Jin C Zhang J Xu M Xu W Sun Z . Msc-derived extracellular vesicle-delivered l-pgds inhibit gastric cancer progression by suppressing cancer cell stemness and stat3 phosphorylation. Stem Cells Int. (2022) 2022:9668239. doi: 10.1155/2022/9668239 35087591 PMC8789473

[65] Zhang J Liu S Zhang J Feng M Chen S Zhang Y . Helicobacter pylori induced mir-362-5p upregulation drives gastric cancer progression and links hepatocellular carcinoma through an exosome-dependent pathway. Front Cell Infect Microbiol. (2025) 15:1582131. doi: 10.3389/fcimb.2025.1582131 40406521 PMC12095252

[66] Tang H Zhong Y Wang J Meng S Yu D Fan B . N2 neutrophils induce cell stemness reprogramming to promote gastric cancer progression via exosomal mirnas. Cell Signal. (2025) 136:112085. doi: 10.1016/j.cellsig.2025.112085 40865592

[67] Zhou Y Li R . Exosomal mir-502-5p suppresses the progression of gastric cancer by repressing angiogenesis through the wnt/β-catenin pathway. Ir J Med Sci. (2024) 193:2681–94. doi: 10.1007/s11845-024-03789-0 39325329

[68] Jing X Xie M Ding K Xu T Fang Y Ma P . Exosome-transmitted mir-769-5p confers cisplatin resistance and progression in gastric cancer by targeting casp9 and promoting the ubiquitination degradation of p53. Clin Transl Med. (2022) 12:e780. doi: 10.1002/ctm2.780 35522909 PMC9076018

[69] Liu X Lu Y Xu Y Hou S Huang J Wang B . Exosomal transfer of mir-501 confers doxorubicin resistance and tumorigenesis via targeting of blid in gastric cancer. Cancer Lett. (2019) 459:122–34. doi: 10.1016/j.canlet.2019.05.035 31173853

[70] Wei W Li J Huang J Jiang Q Lin C Hu R . Exosomal mir-3681-3p from m2-polarized macrophages confers cisplatin resistance to gastric cancer cells by targeting mlh1. Mol Med Rep. (2025) 31(4):94. doi: 10.3892/mmr.2025.13459 39981936 PMC11851060

[71] Gao H Ma J Cheng Y Zheng P . Exosomal transfer of macrophage-derived mir-223 confers doxorubicin resistance in gastric cancer. OncoTargets Ther. (2020) 13:12169–79. doi: 10.2147/ott.S283542 33268995 PMC7701146

[72] Zheng P Chen L Yuan X Luo Q Liu Y Xie G . Exosomal transfer of tumor-associated macrophage-derived mir-21 confers cisplatin resistance in gastric cancer cells. J Exp Clin Cancer Res CR. (2017) 36:53. doi: 10.1186/s13046-017-0528-y 28407783 PMC5390430

[73] Zhou Y Sun YC Zhang QY Wang J Zhu XY Su XY . Tumor-associated macrophage-derived exosome mir-194 confers cisplatin resistance in gc cells. Eur J Med Res. (2025) 30:75. doi: 10.1186/s40001-025-02329-5 39905492 PMC11792191

[74] Liu F Bu Z Zhao F Xiao D . Increased t-helper 17 cell differentiation mediated by exosome-mediated microrna-451 redistribution in gastric cancer infiltrated t cells. Cancer Sci. (2018) 109:65–73. doi: 10.1111/cas.13429 29059496 PMC5765284

[75] Wang J Guan X Zhang Y Ge S Zhang L Li H . Exosomal mir-27a derived from gastric cancer cells regulates the transformation of fibroblasts into cancer-associated fibroblasts. Cell Physiol Biochem Int J Exp Cell Physiol Biochemistry Pharmacol. (2018) 49:869–83. doi: 10.1159/000493218 30184548

[76] Kong Z Zhang M Yuan H Liu J Sang H Zhao P . Exo-mir-1911-5p regulates ferroptosis to promote macrophages m2 polarization-mediated gastric cancer cisplatin resistance via myb/akr1b10/acc. Commun Biol. (2025) 8:1054. doi: 10.1038/s42003-025-08441-w 40664751 PMC12263902

[77] Ye Z Yi J Jiang X Shi W Xu H Cao H . Gastric cancer-derived exosomal let-7 g-5p mediated by serpine1 promotes macrophage m2 polarization and gastric cancer progression. J Exp Clin Cancer Res CR. (2025) 44:2. doi: 10.1186/s13046-024-03269-4 39748408 PMC11694445

[78] Yi J Ye Z Xu H Zhang H Cao H Li X . Egcg targeting stat3 transcriptionally represses plxnc1 to inhibit m2 polarization mediated by gastric cancer cell-derived exosomal mir-92b-5p. Phytomed Int J Phytotherapy Phytopharmacol. (2024) 135:156137. doi: 10.1016/j.phymed.2024.156137 39566403

[79] Zhu T Hu Z Wang Z Ding H Li R Wang J . Microrna-301b-3p from mesenchymal stem cells-derived extracellular vesicles inhibits txnip to promote multidrug resistance of gastric cancer cells. Cell Biol Toxicol. (2023) 39:1923–37. doi: 10.1007/s10565-021-09675-0 35246762

[80] Wang M Zhao C Shi H Zhang B Zhang L Zhang X . Deregulated micrornas in gastric cancer tissue-derived mesenchymal stem cells: novel biomarkers and a mechanism for gastric cancer. Br J Cancer. (2014) 110:1199–210. doi: 10.1038/bjc.2014.14 24473397 PMC3950864

[81] Tang CW Yang JH Qin JW Wu HJ Cui HP Ge LY . Regulation of the pd-1/pd-l1 axis and nk cell dysfunction by exosomal mir-552-5p in gastric cancer. Digestive Dis Sci. (2024) 69:3276–89. doi: 10.1007/s10620-024-08536-0 39020183 PMC11415408

[82] Wang Y Zhang J Shi H Wang M Yu D Fu M . M2 tumor-associated macrophages-derived exosomal malat1 promotes glycolysis and gastric cancer progression. Advanced Sci (Weinheim Baden-Wurttemberg Germany). (2024) 11:e2309298. doi: 10.1002/advs.202309298 38639382 PMC11199979

[83] Xin L Zhou LQ Liu C Zeng F Yuan YW Zhou Q . Transfer of lncrna crnde in tam-derived exosomes is linked with cisplatin resistance in gastric cancer. EMBO Rep. (2021) 22:e52124. doi: 10.15252/embr.202052124 34647680 PMC8647143

[84] Zheng X Xiao H Liu X Huang T Deng C . Exosomal circkiaa1797 regulates cell progression and glycolysis by targeting mir-4429/pbx3 pathway in gastric cancer. Biochem Genet. (2024) 62:1762–78. doi: 10.1007/s10528-023-10529-z 37730964

[85] Zhao H Jiang R Zhang C Feng Z Wang X . Lncrna h19-rich extracellular vesicles derived from gastric cancer stem cells facilitate tumorigenicity and metastasis via mediating intratumor communication network. J Transl Med. (2023) 21:238. doi: 10.1186/s12967-023-04055-0 37005676 PMC10067256

[86] Wang L Bo X Yi X Xiao X Zheng Q Ma L . Exosome-transferred linc01559 promotes the progression of gastric cancer via pi3k/akt signaling pathway. Cell Death Dis. (2020) 11:723. doi: 10.1038/s41419-020-02810-5 32895368 PMC7477231

[87] Chen X Zhang S Du K Zheng N Liu Y Chen H . Gastric cancer-secreted exosomal x26nt increases angiogenesis and vascular permeability by targeting ve-cadherin. Cancer Sci. (2021) 112:1839–52. doi: 10.1111/cas.14740 33205567 PMC8088954

[88] Li S Li J Zhang H Zhang Y Wang X Yang H . Gastric cancer derived exosomes mediate the delivery of circrna to promote angiogenesis by targeting mir-29a/vegf axis in endothelial cells. Biochem Biophys Res Commun. (2021) 560:37–44. doi: 10.1016/j.bbrc.2021.04.099 33965787

[89] Zhang H Wang Y Bai M Wang J Zhu K Liu R . Exosomes serve as nanoparticles to suppress tumor growth and angiogenesis in gastric cancer by delivering hepatocyte growth factor sirna. Cancer Sci. (2018) 109:629–41. doi: 10.1111/cas.13488 29285843 PMC5834801

[90] Yu D Chang Z Liu X Chen P Zhang H Qin Y . Macrophage-derived exosomes regulate gastric cancer cell oxaliplatin resistance by wrapping circ 0008253. Cell Cycle (Georgetown Tex). (2023) 22:705–17. doi: 10.1080/15384101.2022.2146839 36416404 PMC9980452

[91] Jiang L Zhang Y Guo L Liu C Wang P Ren W . Exosomal microrna-107 reverses chemotherapeutic drug resistance of gastric cancer cells through hmga2/mtor/p-gp pathway. BMC Cancer. (2021) 21:1290. doi: 10.1186/s12885-021-09020-y 34856955 PMC8638432

[92] Yong X Mu D Ni H Wang X Zhang T Chang X . Regulation of the cd8^+^ t cell and pdl1/pd1 axis in gastric cancer: unraveling the molecular landscape. Crit Rev Oncol/Hematol. (2025) 212:104750. doi: 10.1016/j.critrevonc.2025.104750 40306470

[93] He W Zhang H Han F Chen X Lin R Wang W . Cd155t/tigit signaling regulates cd8(+) t-cell metabolism and promotes tumor progression in human gastric cancer. Cancer Res. (2017) 77:6375–88. doi: 10.1158/0008-5472.can-17-0381 28883004

[94] Su J Mao X Wang L Chen Z Wang W Zhao C . Lactate/gpr81 recruits regulatory t cells by modulating cx3cl1 to promote immune resistance in a highly glycolytic gastric cancer. Oncoimmunology. (2024) 13:2320951. doi: 10.1080/2162402x.2024.2320951 38419759 PMC10900271

[95] Ye J Huang Q Xu J Huang J Wang J Zhong W . Targeting of glutamine transporter asct2 and glutamine synthetase suppresses gastric cancer cell growth. J Cancer Res Clin Oncol. (2018) 144:821–33. doi: 10.1007/s00432-018-2605-9 29435734 PMC5916984

[96] Li F Sun Y Huang J Xu W Liu J Yuan Z . Cd4/cd8 + t cells, dc subsets, foxp3, and ido expression are predictive indictors of gastric cancer prognosis. Cancer Med. (2019) 8:7330–44. doi: 10.1002/cam4.2596 31631566 PMC6885892

[97] Horozoglu C Hakan MT Sonmez D Yildiz A Demirkol S Aktas F . Ido1-mediated catabolism of tryptophan in gastric tumors: its potential role in the axis of histopathology, differentiation and metastasis. Pathol Res Pract. (2024) 263:155655. doi: 10.1016/j.prp.2024.155655 39442224

[98] Chen W Liang X Peterson AJ Munn DH Blazar BR . The indoleamine 2,3-dioxygenase pathway is essential for human plasmacytoid dendritic cell-induced adaptive t regulatory cell generation. J Immunol (Baltimore Md 1950). (2008) 181:5396–404. doi: 10.4049/jimmunol.181.8.5396 18832696 PMC2614675

[99] Hinata M Kunita A Abe H Morishita Y Sakuma K Yamashita H . Exosomes of epstein-barr virus-associated gastric carcinoma suppress dendritic cell maturation. Microorganisms. (2020) 8(11):1776. doi: 10.3390/microorganisms8111776 33198173 PMC7697542

[100] Herber DL Cao W Nefedova Y Novitskiy SV Nagaraj S Tyurin VA . Lipid accumulation and dendritic cell dysfunction in cancer. Nat Med. (2010) 16:880–6. doi: 10.1038/nm.2172 20622859 PMC2917488

[101] Pacella I Procaccini C Focaccetti C Miacci S Timperi E Faicchia D . Fatty acid metabolism complements glycolysis in the selective regulatory T cell expansion during tumor growth. PNAS. (2018) 115:E6546–e55. doi: 10.1073/pnas.1720113115 29941600 PMC6048537

[102] Lim SA Wei J Nguyen TM Shi H Su W Palacios G . Lipid signalling enforces functional specialization of T(Reg) cells in tumours. Nature. (2021) 591:306–11. doi: 10.1038/s41586-021-03235-6 33627871 PMC8168716

[103] Ma X Xiao L Liu L Ye L Su P Bi E . Cd36-mediated ferroptosis dampens intratumoral Cd8(+) T cell effector function and impairs their antitumor ability. Cell Metab. (2021) 33:1001–12.e5. doi: 10.1016/j.cmet.2021.02.015 33691090 PMC8102368

[104] Liu T Han C Fang P Ma Z Wang X Chen H . Cancer-associated fibroblast-specific Lncrna Linc01614 enhances glutamine uptake in lung adenocarcinoma. J Hematol Oncol. (2022) 15:141. doi: 10.1186/s13045-022-01359-4 36209111 PMC9548164

[105] Shi H Huang S Qin M Xue X Guo X Jiang L . Exosomal Circ_0088300 derived from cancer-associated fibroblasts acts as a Mir-1305 sponge and promotes gastric carcinoma cell tumorigenesis. Front Cell Dev Biol. (2021) 9:676319. doi: 10.3389/fcell.2021.676319 34124064 PMC8188357

[106] Qu X Liu B Wang L Liu L Zhao W Liu C . Loss of cancer-associated fibroblast-derived exosomal Dact3-As1 promotes Malignant transformation and ferroptosis-mediated oxaliplatin resistance in gastric cancer. Drug Resistance Updates: Rev Commentaries Antimicrobial Anticancer Chemotherapy. (2023) 68:100936. doi: 10.1016/j.drup.2023.100936 36764075

[107] Yan W Wu X Zhou W Fong MY Cao M Liu J . Cancer-cell-secreted exosomal Mir-105 promotes tumour growth through the Myc-dependent metabolic reprogramming of stromal cells. Nat Cell Biol. (2018) 20:597–609. doi: 10.1038/s41556-018-0083-6 29662176 PMC5920728

[108] Li M He L Zhu J Zhang P Liang S . Targeting tumor-associated macrophages for cancer treatment. Cell Bioscience. (2022) 12:85. doi: 10.1186/s13578-022-00823-5 35672862 PMC9172100

[109] Wang C Fan X Sun X Xu Y Sun Y Liu J . Tumor associated macrophages in gastric cancer dual roles in immune evasion and clinical implications for targeted therapy. Front Immunol. (2025) 16:1706744. doi: 10.3389/fimmu.2025.1706744 41459539 PMC12738370

[110] Xu J Fang S Dong X Liang C Yang R Zhao Y . Hypoxia-induced upregulation of Hif1a-As3 promotes Msc transition to cancer-associated fibroblasts and confers drug resistance in gastric cancer. Drug Resistance Updates: Rev Commentaries Antimicrobial Anticancer Chemotherapy. (2025) 82:101275. doi: 10.1016/j.drup.2025.101275 40706422

[111] Ji R Zhang B Zhang X Xue J Yuan X Yan Y . Exosomes derived from human mesenchymal stem cells confer drug resistance in gastric cancer. Cell Cycle (Georgetown Tex). (2015) 14:2473–83. doi: 10.1080/15384101.2015.1005530 26091251 PMC4613597

[112] Gu H Ji R Zhang X Wang M Zhu W Qian H . Exosomes derived from human mesenchymal stem cells promote gastric cancer cell growth and migration via the activation of the Akt pathway. Mol Med Rep. (2016) 14:3452–8. doi: 10.3892/mmr.2016.5625 27513187

[113] Qi J Zhou Y Jiao Z Wang X Zhao Y Li Y . Exosomes derived from human bone marrow mesenchymal stem cells promote tumor growth through Hedgehog signaling pathway. Cell Physiol Biochemistry: Int J Exp Cell Physiol Biochemistry Pharmacol. (2017) 42:2242–54. doi: 10.1159/000479998 28817816

[114] Shen Y Xue C Li X Ba L Gu J Sun Z . Effects of gastric cancer cell-derived exosomes on the immune regulation of mesenchymal stem cells by the Nf-Kb signaling pathway. Stem Cells Dev. (2019) 28:464–76. doi: 10.1089/scd.2018.0125 30717632

[115] Tang Y Shen Y Zang X Wu P Li L Qian H . Neutrophil membrane engineered human umbilical cord Msc-derived Sevs enhance anti-tumor efficacy for gastric cancer via delivering Pentraxin 3. J Controlled Release: Off J Controlled Release Soc. (2025) 383:113828. doi: 10.1016/j.jconrel.2025.113828 40345624

[116] Li S Han H Yang K Li X Ma L Yang Z . Exosome-mediated metabolic reprogramming: Effects on thyroid cancer progression and tumor microenvironment remodeling. Mol Cancer. (2025) 24:247. doi: 10.1186/s12943-025-02470-z 41057823 PMC12505858

[117] Zeng H Wang J Xu B Deng H Peng M Deng R . Exosomal Pd-L1 promotes the formation of an immunosuppressive microenvironment in gastric diffuse large B-cell lymphoma. Oncol Rep. (2023) 49(5):88. doi: 10.3892/or.2023.8525 36928140 PMC10073407

[118] Shen Y Lin J Jiang T Shen X Li Y Fu Y . Gc-derived exosomal Circman1a2 promotes cancer progression and suppresses T-cell antitumour immunity by inhibiting Fbxw11-mediated Sfpq degradation. J Exp Clin Cancer Research: CR. (2025) 44:24. doi: 10.1186/s13046-025-03288-9 39856764 PMC11762487

[119] Li J Sun L Chen Y Zhu J Shen J Wang J . Gastric cancer-derived exosomal Mir-135b-5p impairs the function of Vγ9vδ2 T cells by targeting specificity protein 1. Cancer Immunology Immunotherapy: CII. (2022) 71:311–25. doi: 10.1007/s00262-021-02991-8 34159436 PMC10992495

[120] Kimura T Takagane K Itoh G Kuriyama S Meguro K Koyota S . Extracellular vesicles of cancer cells induce Foxp3+ fibroblasts and facilitate tumor invasion via the Wnt3-B-catenin pathway. Oncogene. (2025) 44:4101–13. doi: 10.1038/s41388-025-03552-4 40926045

[121] Wang J Huang F Shi Y Zhang Q Xu S Yao Y . Rp11-323n12.5 promotes the Malignancy and immunosuppression of human gastric cancer by increasing Yap1 transcription. Gastric Cancer: Off J Int Gastric Cancer Assoc Japanese Gastric Cancer Assoc. (2021) 24:85–102. doi: 10.1007/s10120-020-01099-9 32623586

[122] Wei J Wang X Dong D Ru Y Chen L Cheng X . Extracellular vesicle-derived Lncrna-Gc1 serves as a novel biomarker for predicting and monitoring the immunotherapeutic outcomes of patients with gastric cancer. BMC Med. (2025) 23:499. doi: 10.1186/s12916-025-04270-0 40866962 PMC12392478

[123] Sathe A Grimes SM Lau BT Chen J Suarez C Huang RJ . Single-cell genomic characterization reveals the cellular reprogramming of the gastric tumor microenvironment. Clin Cancer Research: Off J Am Assoc For Cancer Res. (2020) 26:2640–53. doi: 10.1158/1078-0432.ccr-19-3231 32060101 PMC7269843

[124] Huang YK Wang M Sun Y Di Costanzo N Mitchell C Achuthan A . Macrophage spatial heterogeneity in gastric cancer defined by multiplex immunohistochemistry. Nat Commun. (2019) 10:3928. doi: 10.1038/s41467-019-11788-4 31477692 PMC6718690

[125] Zhang X Ren B Liu B Wang R Li S Zhao Y . Single-cell Rna sequencing and spatial transcriptomics reveal the heterogeneity and intercellular communication of cancer-associated fibroblasts in gastric cancer. J Transl Med. (2025) 23:344. doi: 10.1186/s12967-025-06376-8 40102930 PMC11917039

[126] Liu Y Sun L Li Y Holmes C . Mesenchymal stromal/stem cell tissue source and *in vitro* expansion impact extracellular vesicle protein and Mirna compositions as well as angiogenic and immunomodulatory capacities. J Extracell Vesicles. (2024) 13:e12472. doi: 10.1002/jev2.12472 39092563 PMC11294870

[127] The Cancer Genome Atlas Research Network. Comprehensive molecular characterization of gastric adenocarcinoma. Nature. (2014) 513:202–9. doi: 10.1038/nature13480 25079317 PMC4170219

[128] Lauren P . The two histological main types of gastric carcinoma: Diffuse and so-called intestinal-type carcinoma. An attempt at a histo-clinical classification. Acta Pathologica Microbiologica Scandinavica. (1965) 64:31–49. doi: 10.1111/apm.1965.64.1.31 14320675

[129] Welsh JA Goberdhan DCI O'Driscoll L Buzas EI Blenkiron C Bussolati B . Minimal information for studies of extracellular vesicles (Misev2023): From basic to advanced approaches. J Extracell Vesicles. (2024) 13:e12404. doi: 10.1002/jev2.12404 38326288 PMC10850029

[130] Wada Y Nishi M Takasu C Tokunaga T Kashihara H Yoshimoto T . A liquid biopsy assay of exosomal Mirna for non-invasive identification of lymph node metastasis in early gastric cancer. Ann Surg Oncol. (2025) 32:10213–23. doi: 10.1245/s10434-025-18088-w 40892283

[131] Wei S Peng L Yang J Sang H Jin D Li X . Exosomal transfer of Mir-15b-3p enhances tumorigenesis and Malignant transformation through the Dynlt1/Caspase-3/Caspase-9 signaling pathway in gastric cancer. J Exp Clin Cancer Research: CR. (2020) 39:32. doi: 10.1186/s13046-019-1511-6 32039741 PMC7011526

[132] Zhang C Zhao WH Zuo D Zhou T Hou W Wang L . Ai-guided Sers defines a pan-cancer diagnostic biomarker. Advanced Sci. (2026) 13(7):e16268. doi: 10.1002/advs.202516268 41243246 PMC12866722

[133] Nguyen KT Xia J Chang C Ghosh A Hade MD Rima XY . Extracellular particles to track cancer biomarkers from tissue to biofluids. J Dent Res. (2026) 105:120–9. doi: 10.1177/00220345251397369 41320881 PMC12968403

[134] Escudé Martinez de Castilla P Tong L Huang C Sofias AM Pastorin G Chen X . Extracellular vesicles as a drug delivery system: A systematic review of preclinical studies. Adv Drug Delivery Rev. (2021) 175:113801. doi: 10.1016/j.addr.2021.05.011 34015418

[135] Yang J Zhang X Cao J Xu P Chen Z Wang S . Circular Rna Ube2q2 promotes Malignant progression of gastric cancer by regulating signal transducer and activator of transcription 3-mediated autophagy and glycolysis. Cell Death Dis. (2021) 12:910. doi: 10.1038/s41419-021-04216-3 34611143 PMC8492724

[136] Mulcahy LA Pink RC Carter DR . Routes and mechanisms of extracellular vesicle uptake. J Extracell Vesicles. (2014) 3:24641. doi: 10.3402/jev.v3.24641 25143819 PMC4122821

[137] Zhu D Zhang T Li Y Huang C Suo M Xia L . Tumor-derived exosomes co-delivering aggregation-induced emission luminogens and proton pump inhibitors for tumor glutamine starvation therapy and enhanced type-I photodynamic therapy. Biomaterials. (2022) 283:121462. doi: 10.1016/j.biomaterials.2022.121462 35272223

[138] Yan HHN Siu HC Law S Ho SL Yue SSK Tsui WY . A comprehensive human gastric cancer organoid biobank captures tumor subtype heterogeneity and enables therapeutic screening. Cell Stem Cell. (2018) 23:882–97.e11. doi: 10.1016/j.stem.2018.09.016 30344100

[139] Zhao Y Li S Zhu L Huang M Xie Y Song X . Personalized drug screening using patient-derived organoid and its clinical relevance in gastric cancer. Cell Rep Med. (2024) 5:101627. doi: 10.1016/j.xcrm.2024.101627 38964315 PMC11293329

[140] Van Deun J Mestdagh P Agostinis P Akay Ö Anand S Anckaert J . Ev-Track: Transparent reporting and centralizing knowledge in extracellular vesicle research. Nat Methods. (2017) 14:228–32. doi: 10.1038/nmeth.4185 28245209

